# Exploring the bi-directional relationship between periodontitis and dyslipidemia: a comprehensive systematic review and meta-analysis

**DOI:** 10.1186/s12903-023-03668-7

**Published:** 2024-04-29

**Authors:** Wanting Ma, Zhaolei Zou, Lisa Yang, Dongjia Lin, Junyi Guo, Zhongyan Shan, Qiannan Hu, Zhi Wang, Bin Li, Juan Fang

**Affiliations:** 1 Hospital of Stomatology, Guanghua School of Stomatology, Guangdong Provincial Key Laboratory of Stomatology, Sun Yat-Sen University, 56 Lingyuan Road West, Guangzhou, 510055 Guangdong China; 2 Central Laboratory, No. 1, Affiliated Hospital of Yunnan University of Traditional Chinese Medicine, Yunnan University of Traditional Chinese Medicine, Yunnan, 650021 Kunming China; 3 Department of Stomatology, Binzhou Medical University Hospital, Binzhou Medical University Hospital, 661 Huanghe Second Road, Shandong, 256603 Binzhou China; 4 Clinical Trials Unit, First Affiliated Hospital of Sun Yat-Sen University, Guangzhou, No. 58, Zhongshan Er Road, Guangzhou, Guangdong 510080 People’s Republic of China

**Keywords:** Dyslipidemia, Periodontitis, Periodontal treatment, Blood lipid treatment, Meta-analysis

## Abstract

**Aim:**

As periodontitis and dyslipidemia are diseases that occur with high incidence, the relationship between them has attracted much attention. Previous studies on these diseases have tended to focus on lipid parameters and periodontitis, we aimed to investigate the relationship between dyslipidemia and periodontitis.

**Materials and methods:**

A comprehensive search to identify the studies investigating the relationship between dyslipidemia and periodontitis was performed on PubMed, Web of Science and Cochrane Library before the date of August, 2023. Studies were considered eligible if they contained data on abnormal blood lipid parameters and periodontitis. Studies that reported mean differences and 95% confidence intervals or odds ratios were used.

**Results:**

A total of 73 publications were included in the meta-analysis. Hyper total cholesterol (TC), triglycerides (TGs), low-density lipoprotein (LDL), very low-density lipoprotein (VLDL) and lower high-density lipoprotein (HDL) levels are risk factors for periodontitis. Periodontal disease is a risk factor for high TG and low HDL levels. Three months after periodontal treatment, the levels of TC, TG and HDL were significantly improved, and statin treatment only improved gingival index (GI) levels compared to that of the dietary control.

**Conclusions:**

The findings reported here suggest that the mutual promotion of periodontitis and dyslipidemia can be confirmed. Non-surgical periodontal therapy may improve lipid abnormalities. It can’t be demonstrated whether systematic application of statins have a better effect on the improvement in periodontal status in patients with dyslipidemia compared to that of the control.

**Supplementary Information:**

The online version contains supplementary material available at 10.1186/s12903-023-03668-7.

## Introduction

Periodontitis involves inflammation that extends deep into tissues and causes loss of supporting connective tissue and alveolar bone [1]. The term ‘periodontal diseases’ encompasses a wide variety of chronic inflammatory conditions involving the gingiva (or gums, which are the soft tissue surrounding the teeth), bone and ligament (the connective tissue collagen fibres that anchor a tooth to alveolar bone) that support teeth [2]. In 2017, the age-standardized prevalence of severe periodontitis was 9.8%, and the number of prevalent cases was 796 million [3]. Gum recession and alveolar bone resorption are typical manifestations of periodontal disease (Pd). Severe periodontitis causes bleeding gums, impaired chewing, and eventually tooth loss. Epidemiologically, periodontitis is associated with several chronic disorders, such as cardiovascular disease, type 2 diabetes mellitus (T2DM), rheumatoid arthritis, inflammatory bowel disease (IBD), Alzheimer’s disease, nonalcoholic fatty liver disease and certain cancers [4]. Multiple parameters, including probing depth (PD), clinical attachment level (CAL), and bleeding on probing (BOP) must be recorded at six locations per tooth to accurately diagnose periodontitis [2]. The plaque index (PI) and gingival index (GI) are also important indicators. The critical risk factor for periodontitis is subgingival plaque. The development of periodontitis is associated with a subgingival microbial community that is imbalanced and enriched with species such as *Porphyromonas gingivalis*, Tannerella forsythia and Treponema denticola. In addition to bacteria, smoking and some systemic diseases, such as diabetes and osteoporosis, are crucial risk factors for periodontitis [5–7].

Dyslipidemia is a disorder that involves lipoproteins in plasma. Laboratory examination showed elevated TC, elevated TG, elevated LDL, elevated VLDL or reduced HDL. There is now a broad consensus that dyslipidemia is a major risk factor for developing cardiovascular disease (CVD). Dyslipidemia can also contribute to the risk of an ischaemic cerebrovascular accident. Since 2002, Asia has been rapidly urbanized and the dietary habits and lifestyles of people have changed, and the prevalence of dyslipidemia has also increased; a large national survey conducted in 2013–2014 in 163,641 Chinese adults showed that the most common forms of dyslipidemia are low plasma HDL-cholesterol levels (20.4% of the population) and high plasma triglyceride levels (13.8%) [8].

Since the 1990s, the relationship between periodontitis and dyslipidemia has attracted considerable interest due to the damage these diseases cause to human health. However, the conclusions of these studies are not completely consistent. To gain expertise on the current standings of research and clinical implications, we searched multiple databases and identified the following relevant directions of research: 1. The influence of dyslipidemia on periodontitis, 2. The influence of periodontitis on dyslipidemia, 3. The influence of periodontal treatment on dyslipidemia, and 4. The effect of blood lipid treatment on periodontitis. In this review, we produced a comprehensive summary of the connection between periodontitis and dyslipidemia.

## Materials and methods

This review was conducted and reported according to the PRISMA statement [9] and the Cochrane Handbook [10].

### Principal question

Is there an association between dyslipidemia and periodontitis? Will the treatment of dyslipidemia or periodontitis influence the other disease?

### Search strategy

**Fig. 1:**
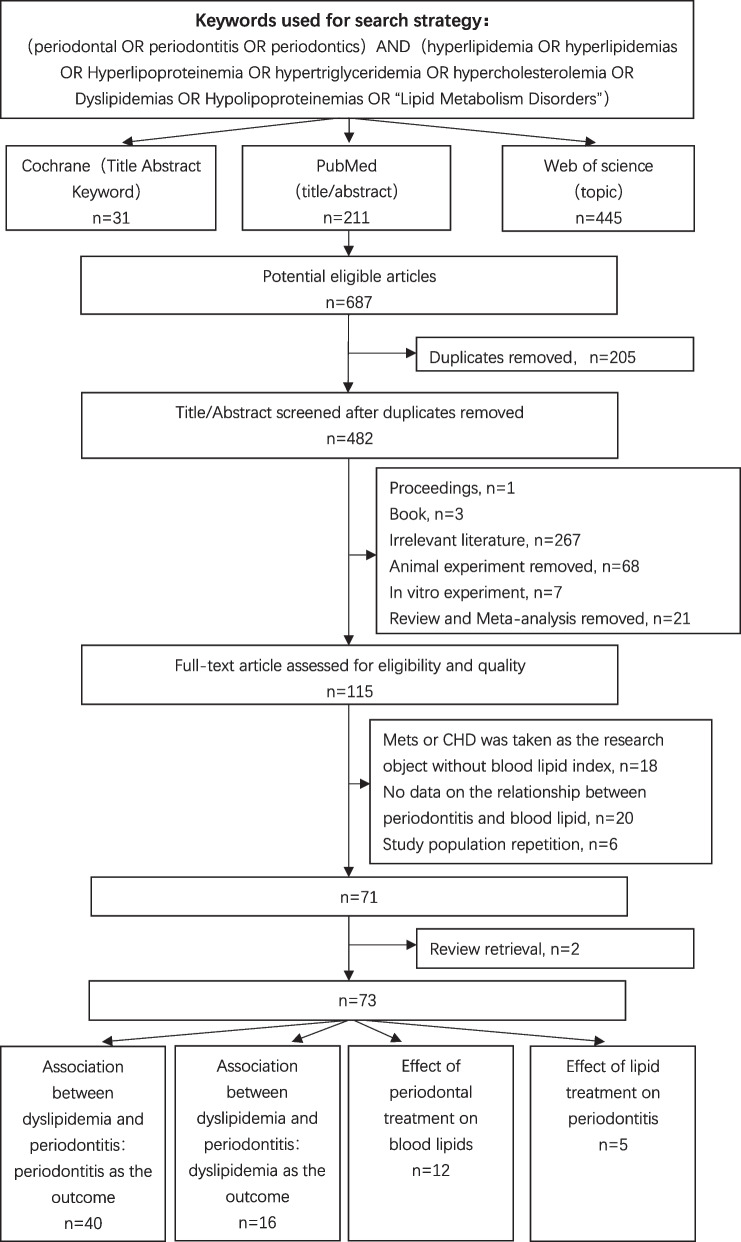
Flow chart of the screening process in this study

The following electronic databases were searched for dates before August 1st, 2023: PubMed, Web of Science and Cochrane Library. The detailed search strategy is shown in Fig. 1.

Two independent reviewers screened records for potentially eligible titles and abstracts and subsequently reviewed full texts to determine the inclusion in the meta-analysis. Disagreements were resolved with a third reviewer to reach a consensus.

### Study selection

Abstracts and references were managed using EndNote. The criteria for selecting the eligible articles were as follows: (I) cross-sectional studies, cohort studies, case–control studies and clinical trials. (II) The main goal was to research the relationship between dyslipidemia and periodontitis. (III) When the study population was repeated, we used the most recent study that involved the largest study population. (IV) Studies on syndromes, such as metabolic syndrome (MetS) or Coronary heart disease (CHD), in which the blood lipids were described but the blood lipid levels were not described were excluded. (V) All in vitro and in vivo animal experiments were excluded.

### Data extraction

We extracted the data on the author, year, country, study design, sample size, diagnosis criteria for periodontal disease, BMI match or correction, mean age, age ratio, sex ratio, matching or correction factor, effect index and quality evaluation. We will list them separately in Tables 1, 2, 3 and 4.

**Table 1:** Main characteristic of the eligible studies for the association between dyslipidemia and periodontitis:periodontitis as the outcome

Author, year	Country	Study design	Sample size (P/ HC)	Diagnosis criteria for periodontitis	Mean age (year)	age Ratio (P: HC)	% Males	Males ratio (P: HC)	Dyslipidemia diagnosis (any of the following indicators, mg/dl)	Lipid Index	Effect index	Matching or adjusted factor	Quality
Al-Otaibi DH, 2008 [11]	Saudi Arabia	Cross-sectional	60/30	Insecure	*NI*	1	*NI*	1.00	*NI*	TC, TG, LDL, HDL	Mean (SD)	Age, gender	Low
Anitha A, 2014 [12]	India	Cross-sectional	25/25	Insecure	*NI*	*NI*	24	0.71	*NI*	HDL, TG	Mean	Age, gender	Moderate
Banihashemrad SA, 2008 [13]	Iran	Cross-sectional	71	Insecure	26.68	*NI*	78.87	*NI*	*NI*	TG, TC	Mean (SD)	*–*	Low
Bullon P, 2014 [14]	Spain	Cross-sectional	13/175	Secure	31.96	1.04	0	–	*NI*	TC, HDL, LDL, VLDL, TG	Mean (SD)	*–*	Moderate
Cury EZ, 2018 [15]	Brazil	Case-control	40/40	Secure	46.25	1.08	65.3	0.74	TG > 200; TC > 200; LDL > 130; HDL < 60	TC, HDL, LDL, TG	Mean (SD), OR	Age, gender	Moderate
Cutler CW, 1999 [16]	Korea	Case-control	26/25	Secure	46.10	1.21	49.02	0.64	*NI*	TG, TC	Mean (SD), OR	Age, gender, race, LPS reactivity, ELISA titer	Moderate
Doraiswamy S, 2017 [17]	India	Case-control	30/30	Secure	43.3	1.06	*NI*	*NI*	TC > 200; TG > 200; HDL > 55; LDL > 130; VLDL> 25–35	TC, HDL, LDL, VLDL	Mean (SD), Post-OR†	Gender, age,	Moderate
Fentoglu O, 2020 [18]	Turkey	Case-control	123/68	Secure	. *NI*	*NI*	*NI*	. *NI*	TG > 200; TC > 200; LDL > 130; HDL < 35; VLDL> 40	TC, TG, LDL, HDL	OR	*–*	High
Gao H, 2015 [19]	China	Case-control	185/138	Secure	27.91	0.96	40.56	1.00	*NI*	TC, HDL, LDL, TG	Mean (SD)	Age, gender, smoking, BMI	Moderate
Golpasand HL, 2014 [20]	Iran	Case-control	45/45	Secure	34.74	0.96	46.67	0.91	TC > 220; TG > 200; LDL > 178; HDL < 29	TC, TG, HDL, LDL	Mean (SD), Post-OR	Age, gender, number of teeth	Moderate
Güler B, 2020 [21]	Turkey	Cross-sectional	LagP: 16; GagP: 16	*NI*	34.79	1.02	25	1.00	*NI*	HDL	Mean (SD)	*–*	Moderate
Hamissi J, 2011 [22]	Iran	Case-control	30/30	Secure	35.32	1.04	*NI*	1.00	*NI*	TC, HDL, LDL, TG	Mean (SD)	Age, gender	Moderate
Han SJ, 2019 [23]	Korea	Cross-sectional	4997/12007	Insecure	44.61	1.29	50.2	1.28	TC ≥ 240; HDL-C < 40; HDL-C ≥ 60; TG ≥ 200; LDL-C ≥ 160	TC, HDL, LDL, TG	OR	Adjust 1	Moderate
Kalburgi V, 2014 [24]	India	Case-control	40/20	Insecure	*NI*	*NI*	55	1.15	*NI*	TC, HDL, LDL, TG	Mean (SD)	*–*	Moderate
Kim, SR, 2020 [25]	Korea	Cross-sectional	14,608	Insecure	*NI*	*NI*	*NI*	*NI*	TC > 240; TG > 200	TC,TG	OR	Adjust 2	Moderate
Koshy BS, 2017 [26]	India	Cross-sectional	50/25	Secure	40.49	1.14	56	0.90	*NI*	TC, TG, HDL, LDL, VLDL	Mean (SD)	*–*	Moderate
Kumar KR, 2014 [27]	India	Cross-sectional	25/25	Secure	*NI*	*NI*	*NI*	*NI*	*NI*	LDL,HDL, TG	Mean (SD)	*–*	Low
Kushiyama M, 2009 [28]	Japan	Cross-sectional	316/754	Secure	*NI*	*NI*	26.26	*NI*	HDL-C < 40 for males < 50 for females; TG ≥ 150	TC, TG, HDL	Mean (range), OR	Age, gender, smoking habits	Moderate
Lee JB, 2013 [29]	Korea	Cross-sectional	5558/9976	Insecure	44.20	1.28	42.55	0.63	TC ≥ 240; TG > 200; HDL-C ≤ 40; LDL ≥ 160	TC, TG, HDL	OR	Adjust 3	Moderate
Lee S, 2018 [30]	Korea	Cross-sectional	1365/5540	Insecure	*NI*	*NI*	41.68	1.52	TC ≥ 240; HDL-C ≤ 40; HDL-C ≥ 60; LDL-C ≥ 160; TG ≥ 200	TC, HDL, LDL, TG	Mean (95% CI), OR	Adjust 4	Moderate
Losche W, 2000 [31]	Germany	Case-control	39/40	Insecure	55.10	0.99	41.77	1.39	TC > 230; LDL-C > 160; HDL- C > 45; TG > 200	TG, TC, LDL, HDL	Mean (SD), Post-OR	*–*	Moderate
Machado AC, 2005 [32]	Brazil	Case-control	30/30	Insecure	43.80	0.98	56.67	1.00	TC ≥ 240; LDL-C ≥ 160; HDL-C ≤ 35; TG ≥ 200	TG, TC, LDL,HDL	Mean (SD), Post-OR	Age, gender	Moderate
Moeintaghavi A, 2005 [33]	Iran	Case-control	40/40	Insecure	31.90	1.03	61.25	1.04	TC > 220; LDL-C > 190; HDL-C < 29; TG > 200	TC, TG, LDL, HDL	Mean (SD), OR	Age, gender	Moderate
Moghadam SA, 2015 [34]	Iran	Case-control	61/60	Insecure	*NI*	1	50	1.00	*NI*	TG, TC	Mean (SD)	Age, gender	Moderate
Monteiro AM, 2009 [35]	Brazil	Case-control	40/40	Insecure	44.95	1.01	42.5	1.13	TC > 200; LDL > 130; HDL < 40; TG > 150	TC, HDL, LDL, TG	Mean (SD), Post-OR	Age, gender, BMI	High
Nibali L, 2007 [36]	United Kingdom	Case-control	302/183	Secure	40.43	1.03	45.62	1.01	*NI*	TG, TC, LDL, HDL	Mean (95% CI)	Age, gender, smoking ethnicity	Moderate
Penumarthy S, 2013 [37]	India	Case-control	30/30	Secure	33.92	1.46	*NI*	*NI*	TG ≥ 150; TC ≥ 200; HDL-C ≤ 60; LDL-C ≥ 130	TG, TC, LDL, HDL	Mean (SD)	*–*	Low
Sandi RM, 2014 [38]	India	Cross-sectional	40/40	Insecure	44.82	1.05	52.5	*NI*	*NI*	TC, TG, HDL, LDL	Mean (SD)	Age	Moderate
Saxlin T, 2008 [39]	Finland	Cross-sectional	1297	Insecure	*NI*	*NI*	39.24	*NI*	*NI*	TG, HDL, LDL	Quintile, RR	Adjust 5	High
Shi D, 2006 [40]	China	Case-control	40/37	Insecure	32.59	1.36	35.06	0.74	*NA*	TC, TG	Mean (SD)	*–*	Moderate
Shimazaki Y, 2007 [41]	Japan	Cross-sectional	37/547	Insecure	55.74	1.07	0	–	TG > 150; HDL-C < 50	TG, HDL	Mean (SD), OR	Age, smoking status	Moderate
Sridhar R, 2009 [42]	India	Case-control	30/30	Secure	44.43	1.09	43.33	1.17	*NI*	HDL,TG	Mean (SD)	*–*	Low
Taleghani F, 2010 [43]	Iran	Cohort (retrospective)	26/26	Secure	46.50	0.94	34.62	1.00	LDL-C > 180; HDL-C < 30; TG > 200; TC > 250	TC, TG, HDL, LDL	Mean (SD), Post-OR	*–*	Moderate
Thapa S, 2016 [44]	United States	Cross-sectional	376/685	Secure	49.90	*NI*	51.84	0.75	TC ≥ 240; LDL ≥ 160; HDL < 50; TG ≥ 200	TC	OR	Adjust 6	Moderate
Thomas B, 2017 [45]	India	Cross-sectional	300	Secure	*NI*	*NI*	*NI*	*NI*	*NI*	TC, HDL, LDL, VLDL, TG	Mean (SD)	*–*	Moderate
Wang Y, 2007 [46]	China	Cross-sectional	280/178	Insecure	53.50	*NI*	57.64	*NI*	TC ≥ 220; TG ≥ 150	TC, TG	OR	Age, education, oral hygiene habits,	Moderate
Zhou SY, 2012 [47]	China	Cross-sectional	40/20	Secure	46.50	1.07	33.33	1.17	TC > 220; TG > 150; LDL-C > 120	TC, TG, HDL, LDL	Mean (SD)	*–*	Moderate
Xiaoyuan Guan, 2022 [48]	China	Cross-sectional	397/285	Secure	NI	1.03	57.51	1.80	NI	TG, LDL, HDL	Mean (SD)	Gender, age, BMI, alcohol consumption, exercise frequency, smoking habits	Moderate
Feng Zhang, 2022 [49]	China	Cross-sectional	219	Secure	30.10	NI	0	0	NI	HDL	Mean (SD)	Adjust 7	Moderate
Chunyan Ding, 2023 [50]	China	Cross-sectional	163/57	Insecure	NI	NI	55.00	0.97	TC ≥ 2 mmol/L, TG ≥ 3 mmol/L, HDL-C, < 1.0 mmol/L, LDL-C ≥ 1 mmol/L	TC, TG, LDL, HDL	OR	Age, overweight or obesity, alcohol consumption, cardiovascular history, and hypertension	Moderate

Adjust 1: age, gender, family income, education level, alcohol consumption experience in a lifetime, smoking status, regular walking, fat intake,number of remaining teeth, active caries, diabetic status, obesity, and hypertension

Adjust 2: Age, gender, BMI, education level, family income, marital status, house ownership, number of people living together, health insurance coverage and economic activities

Adjust 3: age, gender, family income, educational level,use of floss,alcohol consumption experience in a lifetime, present smoking status, active caries,diabetes mellitus and obesity

Adjust 4: age, area, education, BMI, alcohol intake, menopausal status (in women),and smoking status

Adjust 5: Gender, age, education level, frequency of tooth brushing, dental visit pattern, presence of plaque, lipid drugs, alcohol consumption, BMI

Adjust 6: Age, gender, income, education level, calcium level, smoking status, DM and BMI

Adjust 7: Age, gestational age, educational level, household monthly income, parity and pathological abortion, BMI, SBP, DBP, glucose, HbA1c, TG, HDL-c, TBA, AST, TP, and Urine ACR using backward stepwise method

*Abbreviation*: *P* periodontitis, *HC* health control, *ABL* alveolar bone loss, *PD* probing depth, *CAL* clinical attachment loss, *TC* total cholesterol, *TG* triglyceride, *LDL* low density lipoprotein cholesterol, *HDL* high density lipoprotein cholesterol, *VLDL* very low density lipoprotein cholesterol, *NI* not informed, *SD* standard error, *OR* odds ratio

† Post-OR was post-hoc calculated using the enough information of the study

**Table 2:** Main characteristic of the eligible studies for the association between dyslipidemia and periodontitis:dyslipidemia as the outcome

Author, year	Country	Study design	Sample size (DLP/HC)	DLP diagnosis (any of the following indicators, mg/dl)	Mean age (year)	Age ratio (DLP:HC)	% Males	Males ratio (DLP:HC)	Periodontal index	Effect index	Matching or adjusted factor	Quality
Almeida AJ, 2013 [51]	Brazil	Cross-sectional	67/57	TG > 150; TC > 200; LDL > 100; HDL < 40 in males and < 50 for females	48.83	1.13	23.39	0.27	PD, CAL	Mean (SD)	Age, DM2, BMI, Smoking	Moderate
Awartani F, 2010 [52]	Saudi Arabia	Cross-sectional	30/30	TG > 200; TC > 200; LDL-C > 130; HDL-C < 35	46.70	1.02	0	*–*	PI, BOP, PPD, CAL	Mean (SD)	Age	Moderate
D’Aiuto F, 2008 [53]	United Kingdom	Cross-sectional	1919/11758	TG > 150; HDL-C < 40 for men and < 50 for women	40.80	*NI*	49.40	*NI*	Mild/Moderate/Severe periodontitis	Mean (95% CI), OR	Adjust 1	Moderate
Dogan B, 2015 [54]	Turkey	Cross-sectional	99/28	NA	*NI*	*NI*	38.33	0.59	PI, GI, PD, CAL, SBI,	Mean (SD)	–	Moderate
Fentoglu O, 2009 [55]	Turkey	Case-control	51/47	TG > 200; TC > 200; LDL > 130; HDL < 35	48.40	1.04	43.87	0.55	PI, PD, BOP, CAL	Mean (SD)	Adjust 2	High
Fentoglu O, 2011 [56]	Turkey	Cross-sectional	123/68	TRG > 200; TC > 200; LDL > 130; HDL < 35; VLDL> 40	43.84	1.02	49.21	1.12	PI, GI, PPD, BOP, CAL	Median (range)	–	Moderate
Fentoglu O, 2015 [57]	Turkey	Case-control	18/19	TG > 200; TC > 200; LDL > 130; HDL < 35; VLDL > 40	43.13	0.99	48.65	1.06	PI, GI, BOP, PD, CAL	Mean (SD)	Age, gender, oral hygiene behaviors	Moderate
Fukui N, 2012 [58]	Japan	Cross-sectional	958/5463	HDL < 40 for males and < 50 for females; TG ≥ 150	43.45	1.07	77.00	1.24	PD, CAL	Mean (IQR), OR	–	Moderate
Katz J, 2002 [59]	Israel	Cross-sectional	10,590	NA	31.00	*NI*	89.00	*NI*	CPITN	Mean (SD)	Age, smoking, BMI, DBP	Moderate
Kemer ES, 2018 [60]	Turkey	Cross-sectional	67	TG > 200; TC > 200; LDL > 130; HDL < 35	*NI*	*NI*	0	*–*	PI, GI, PD, CAL	Mean (SD), Correlation efficient	Age	Moderate
Lutfioglu M, 2017 [61]	Turkey	Case-control	15/15	TC > 200; TG > 200; LDL > 130; HDL < 35	41.89	1.19	46.67	1	PI, GI, BOP, PPD, CAL	Mean (SD)	Age, gender	Moderate
Scardina GA, 2011 [62]	Italy	Case-control	20/20	TC > 200; 160 < LDL < 190	63.07	1.09	42.86	1	Periodontitis	Mean (SD)	–	Low
Shivakumar T, 2013 [63]	India	Case-control	60/60	TG > 200; TC > 200; LDL > 130; HDL < 35	48.40	1.045	43.88	0.55	PI, PD, BOP, CAL	Mean (SD)	–	Low
Yu Z, 2012 [64]	China	Cross-sectional	903	TG ≥ 150; HDL < 40 for male and < 50 for female	62.58	*NI*	50.50	*NI*	No-mild/moderate-severe periodontitis	OR	Adjust 3	Moderate
Haihua Zhu, 2022 [65]	China	Cross-sectional	874	TC ≥ 200; HDL < 40 for male and < 50 for female; LDL > 130; TG ≥ 150	70.43	*NI*	49.90	1.34	No-mild/moderate-severe periodontitis	OR	Adjust 4	Moderate
YunSook Jung, 2023 [66]	United States	Cross-sectional	12,254	TG ≥ 150; HDL < 40 for male and < 50 for female;	*NI*	48.31	*NI*	*NI*	CAL, PD	OR	Adjust 5	Moderate

Adjust 1: Age, gender, years of education, poverty-to-income ratio, race, general condition, and smoking

Adjust 2: Age, gender, BMI, high blood pressure, number of missing teeth and daily brushing habits

Adjust 3: Age, gender, years of education, economic income, drinking and smoking

Adjust 4: Age, gender, BMI, alcohol drinking frequency (never, seldom, often, everyday), exercise frequency (never, 1–3 times per week, 4–6 times per week, once per day, more than once per day), smoking habits (current smoker; former smoker; nonsmoker)

Adjust 5: Age, sex, race, education, income, missing teeth, smoking, alcohol intake, regularity of dental visits, BMI, and physical activity

*Abbreviation*: *DLP* dyslipidemia, *HC* health control, *ABL* alveolar bone loss, *PD* probing depth, *CAL* clinical attachment loss, *BOP* bleeding on probing, *PI* plaque index, *GI* gingival index, *TC* total cholesterol, *TG* triglyceride, *LDL* low density lipoprotein cholesterol, *HDL* high density lipoprotein cholesterol, *VLDL* very low density lipoprotein cholesterol, *NI* not informed, *DBP* diastolic blood pressure, *SD* standard error, *IQR* interquartile range, OR odds ratio

**Table 3:** Main characteristic of the eligible studies for the periodontitis treatment and dyslipidemia

Author, year	Country	Study design	Sample size (treat / control)	Diagnosis criteria for periodontitis	Mean age (year)	Age ratio (treat: control)	% Males	Male ratio (treat: control)	Therapeutic Schedule	Dyslipidemia diagnosis (any of the following indicators, mg/dl)	Lipids index	Effect index	Matching or correction factor	Quality
Duan JY, 2009 [67]	China	Cohort (prospective)	20	Secure	55.60	*NI*	55	*NI*	Initial therapy	TC > 220; TG > 150; HDL < 35; LDL > 140	TC, TG, LDL, HDL	Mean (SD)	–	High
Fentoglu O, 2010 [68]	Turkey	Cohort (prospective)	20	Insecure	51.85	*NI*	40	*NI*	Initial therapy	TG > 200; TC > 200; LDL > 130; HDL < 35; VLDL > 40	TG, TC, LDL, HDL	Mean (SD)	–	High
Fu YW, 2016 [69]	Chnia	RCT	54/55	Secure	46.93	0.99			Treat group: Initial therapy; Control group: supragingival scaling	TG > 20; HDL < 40; LDL > 160	TC, TG, LDL, HDL	Mean (SD)	–	High risk of bias
Losche W, 2005 [70]	Germany	Cohort (prospective)	32	*NI*	42.80	*NI*	53.13	*NI*	Initial therapy	*NI*	TC, TG, LDL, HDL	Mean (SD)	–	Moderate
Macovei-Surdu A, 2013 [71]	Romania	Cohort (prospective)	30/30	Insecure	39.53	0.96	60	1.36	Treat group: Initial therapy: Control group: oral hygiene maintenance measures	*NI*	TC, TG, LDL, HDL	Mean	–	Moderate
Nassar PO, 2012 [72]	Brazil	RCT	10/10	Secure	*NI*	*NI*	*NI*	*NI*	Treat group: Initial therapy, Maintenance therapy: Control group: Initial therapy	*NI*	TC, TG	Mean (SD)	–	Unclear risk of bias
Nibali L, 2015 [101]	United Kingdom	Cohort (prospective)	12	Secure	*NI*	*NI*	*NI*	*NI*	Initial therapy, open flap debridement		LDL, HDL, IDL, VLDL	Mean (SD)	Age, gender and number of teeth	High
Nicolaiciuc O, 2016 [74]	Romania	Cohort (prospective)	20	Insecure	49.55	*NI*	40	*NI*	Initial therapy	TG > 200; TC > 200; LDL > 130; HDL < 35; VLDL > 40	TG, TC, LDL, HDL, VLDL	Mean (SD)	–	Moderate
Oz SG, 2007 [75]	Turkey	RCT	25/25	Insecure	50.34	0.95	38	1.11	Treat group: Initial therapy: Control group: without applying periodontal treatment	TC>200; LDL>130; HDL<35; VLDL> 40; TG>200	TG, TC, LDL, HDL, VLDL	Mean (SD)	–	Unclear risk of bias
Tawfig A, 2015 [76]	Saudi Arabia	RCT	15/15	Insecure	*NI*	*NI*	63.33	1.38	Treat group: Initial therapy, oral hygiene instructions, Control group: oral hygiene instructions	TC ≥200; LDL ≥130; HDL ≤35; TG ≥150	TC, TG, LDL, HDL	Mean (SD)	–	Unclear risk of bias
Zuza EP, 2016 [77]	Brazil	Cohort (prospective)	54	Secure	44.26	*NI*	25.93	*NI*	Initial therapy	*NI*	TC, TG, LDL, HDL	Mean (SD)	–	High
Abhay Pandurang Kolte, 2022 [78]	India	Intervention trial	60	Secure	NI	*NI*	36.67	*NI*	Non-surgical periodontal treatment	*NI*	TC, TG, LDL, HDL	Mean (SD)	–	–

Initial therapy: supragingival scaling, subgingival scaling and root planning

*Abbreviation*: *RCT* randomised clinical trial, *TC* total cholesterol, *TG* triglyceride, *LDL* low density lipoprotein cholesterol, *HDL* high density lipoprotein cholesterol, *VLDL* very low density lipoprotein cholesterol, *NI* not informed, *SD* standard error

**Table 4:** Main characteristic of the eligible studies for the dyslipidemia treatment and periodontitis

Author, year	Country	Study design	Sample size (Treat / control)	Diagnosis criteria for periodontitis	Treatment	Mean age (year)	Age ratio (Treat / control)	% Males	Males ratio (Treat / control)	Diagnosis criteria for Hyperlipidemia	Research index	Effect index	Matching or correction factor	Quality
Fentoglu O, 2015 [79]	Turkey	Cohort (prospective)	23/29	secure	Statins	44.58	*NI*	50	*NI*	TG > 200; TC > 200; LDL > 130; HDL < 35; VLDL> 40	TC, TG, LDL, HDL, VLDL	Median (range)	–	Moderate
Sangwan A, 2013 [80]	India	Cross-sectional	50/44	*NI*	Simvastatin	43.62	1.10	57.45	1.02	TG > 200; TC > 200; HDL > 130; HDL < 35	PD, CAL, GI, PI	Mean (SD)	–	High
Sangwan A, 2016 [81]	India	Cohort (prospective)	36/36	*NI*	Atorvastatin	43.31	1.06	59.72	1.26	TC > 200; TG > 200; LDL > 130; HDL < 35	PI, PD, CAL, GI	Mean (SD)	Age, gender, BMI	High
Sayar F, 2016 [82]	Iran	Cross-sectional	50/50	*NI*	Simvastatin	47.04	1.00	49	1.33	TC > 200; LDL > 130	PI, CAL, BOP, PD	Mean (SD)	–	High
Sayar F, 2017 [83]	Iran	Cross-sectional	30/30	*NI*	Gemfibrozzi	44.72	1.04	53.33	1.29	Male TG > 160; female TG > 140	CAL, PD, PI, BOP	Mean (SD)	Adjust 1	High

Adjust 1: Age, number of remaining teeth, PI, BMI

*Abbreviation*: *TC* total cholesterol, *TG* triglyceride, *LDL* low density lipoprotein cholesterol, *HDL* high density lipoprotein cholesterol, *VLDL* very low density lipoprotein cholesterol, *NI* not informed, *SD* standard error

### Clinical definitions of periodontal disease

To eliminate the diagnosis bias, we made the following definitions:

- Secure periodontitis:

At least one site with a probing depth (PD) ≥ 4 mm in every quadrant and radiographic evidence of bone loss, or.

At least two sites in non-adjacent teeth with interproximal attachment loss ≥3 mm, or.

At least two sites with PD ≥ 4 mm and CAL ≥ 3 mm, or.

A community periodontal index (CPI) score of 4 in at least one quadrant, or.

For cases in which no CAL or PD is reported, radiographic marginal alveolar bone loss is ≥30%

b. Insecure periodontitis:

Periodontitis was defined only by PD or CAL but without a clear definition.

### Quality assessment

The quality of the included case–control studies and cohort studies was assessed using the Newcastle–Ottawa Scale (NOS). The article quality was assessed as follows: low quality = 0–4; moderate quality = 5–6; high quality = 7–9. The methodological quality of the cross-sectional studies included was assessed using an 11-item checklist recommended by the Agency for Healthcare Research and Quality (AHRQ). An item was scored “0” if it was answered “NO” or “UNCLEAR”; if it was answered “YES”, then the item scored 1″. Article quality was assessed as follows: low quality = 0–3; moderate quality = 4–7; high quality = 8–11 [84]. The quality of the randomized controlled trial was assessed using the Cochrane Collaboration’s tool for assessing the risk of bias in randomized trials. Detailed quality evaluation is listed in Supplementary Tables S1, 2, 3, 4.

### Data analysis

For continuous data, the pooled effect was estimated as the mean difference (MD) and the 95% confidence interval (CI). For the dichotomous data, the pooled effect was estimated as the odds ratio (OR) and 95% CI. All pooled estimates were obtained using the random effects model of DerSimonian and Laird, which considers both within-study and between-study variations and provides more conservative estimates than those of a fixed-effects model [85, 86]. The heterogeneity among studies was assessed using the I^2^ statistic, which determines the proportion of variability across studies that is due to heterogeneity rather than sampling error [87]. A *P* value less than 0.10 or an I^2^ -value over 50% indicates substantial heterogeneity.

If heterogeneity existed in the pooled studies, meta-regressions were performed to explain the sources of between-study heterogeneity, and these sources included the published year, region, study design, total sample size, quality of study, age, sex, BMI matched, periodontal diagnosis and multi-variable analysis.

To examine the influence of each study on the pooled estimates, sensitivity analyses were conducted using the leave-one-out method, which removes one study each time and repeats the analysis [88]. Egger’s and Begg’s tests were used to detect publication bias in all meta-analyses.

All statistical analyses were carried out using R 3.6.1 software. *P* values less than 0.05 were considered statistically significant, except when otherwise specified.

## Results

### Literature search

The literature search identified 687 relevant publications. A total of 205 duplicates were removed. Screening the titles and abstracts resulted in the elimination of 267 studies that failed to meet any of the inclusion criteria, and all proceedings and books were removed. We also excluded all animal experiments and in vitro experiments.Twenty-one reviews and meta-analyses were removed. A total of 115 papers were selected for full-text screening. In 18 articles, Mets or CHD was used as the research object but without a blood lipid index, and 20 articles did not include data on the relationship between periodontitis and blood lipids; we excluded these articles from our current investigation. 6 studies contained repetitive populations, and we used the most recent study with the largest study population. After the quadratic search for reviews, we finally identified 73 articles. (Fig. 1).

### Association between dyslipidemia and periodontitis: periodontitis as the outcome

i. TC and periodontitis

**Fig. 2:**
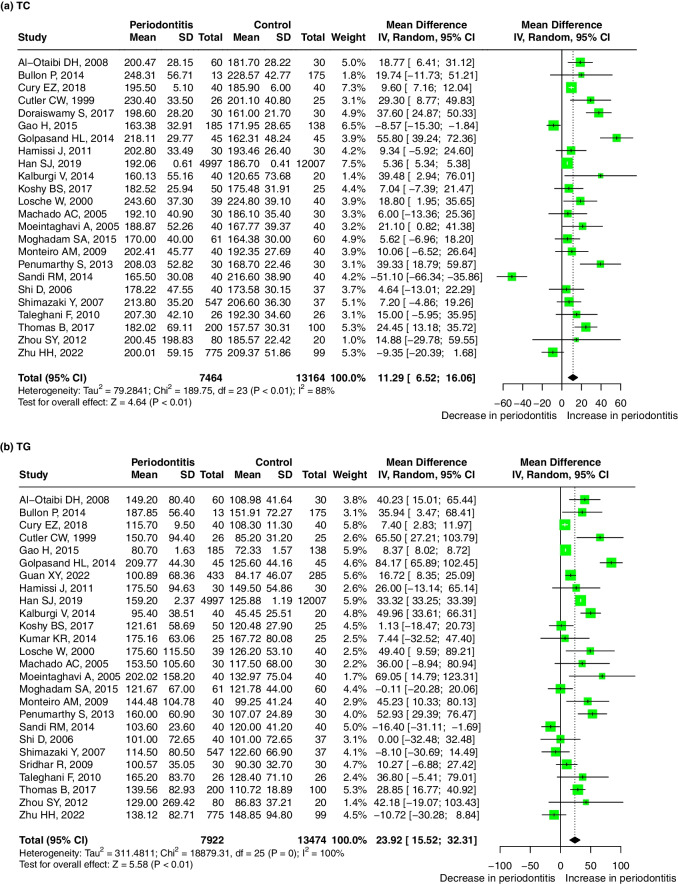

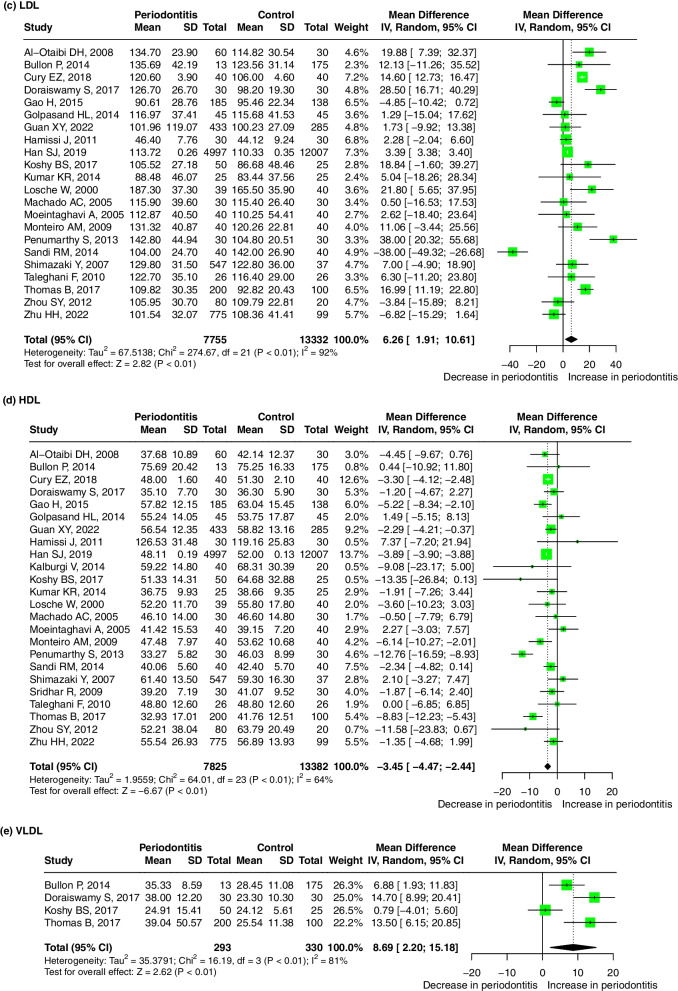
Forest plot of mean difference for comparisons:periodontitis versus non-periodontitis. (**a**) periodontitis have high serum TC level; (**b**) periodontitis have high serum TG level; (**c**) periodontitis have high serum LDL level; (**d**) periodontitis have low serum HDL level; (**e**) periodontitis have high serum VLDL level. Abbreviations: TC:Total cholesterol, TG: triglycerides, LDL: low-density lipoprotein, HDL: high-density lipoprotein, VLDL: very low-density lipoprotein

Twenty-four studies evaluated the difference in the serum TC level between periodontitis and healthy control groups with the mean (SD). TC levels were higher in patients with periodontitis than in controls, with a pooled mean difference of 11.29 mg/dL (95%-CI: 6.52, 16.06, *p* < 0.01). There was significant heterogeneity between the studies (I^2^ = 88%, *p* < 0.01) (Fig. 2a). Meta-regression showed that the sources of the heterogeneity may be diagnosis of periodontitis (P diagnosis) (*p* = 0.041) and BMI matching (*p* = 0.073) (Table S5). The result was robust regardless if any one study was omitted (Supplementary Fig. 1-a). No significant publication bias was found after Egger’s (*p* = 0.148) and Begg’s tests (*p* = 0.275).

**Fig. 3:**
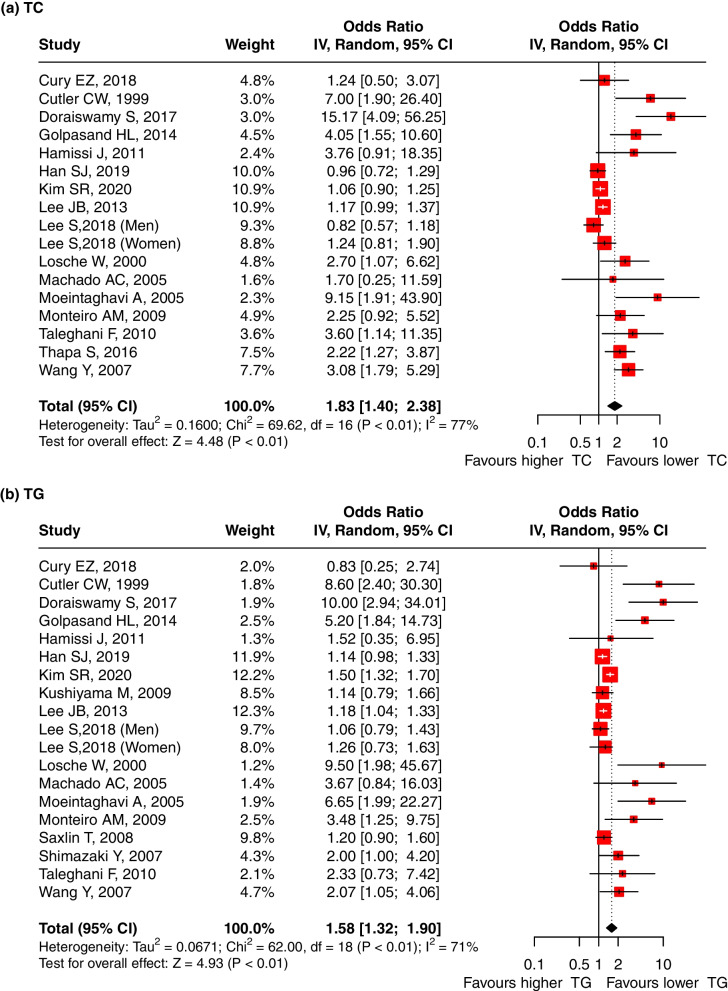

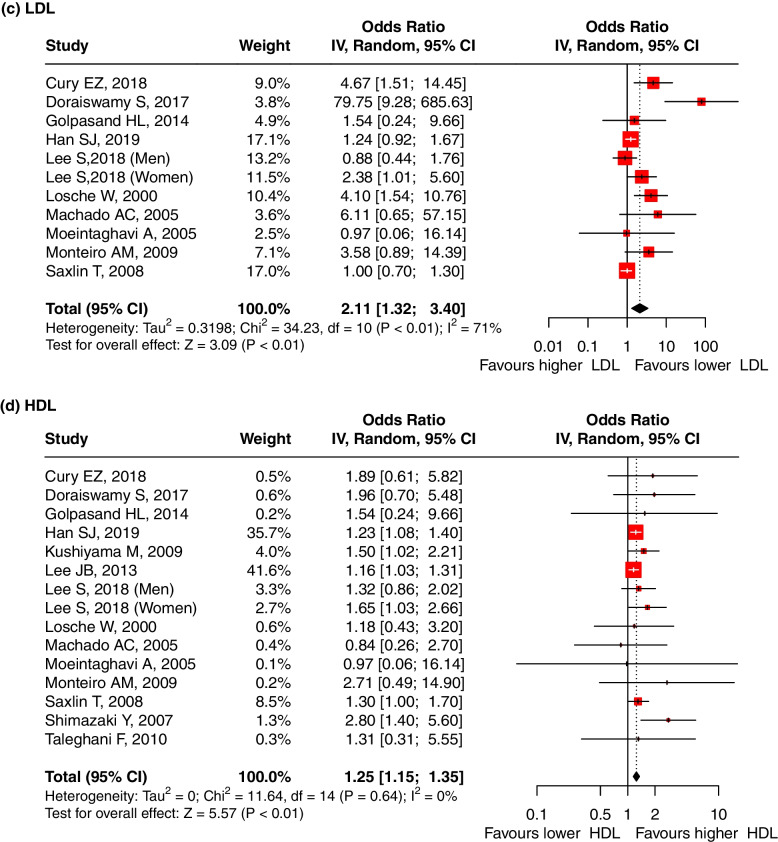
Forest plot of OR values for different lipids levels with the risk of periodontitis. **a** high TC level is associated with the high risk of periodontitis; (**b**) high TG level is associated with the high risk of periodontitis; (**c**) high LDL level is associated with the high risk of periodontitis; (**d**) low HDL level is associated with the high risk of periodontitis. Abbreviations: TC:Total cholesterol, TG: triglycerides, LDL: low-density lipoprotein, HDL: high-density lipoprotein

Sixteen studies reported the OR values to evaluate the association between the serum TC level and periodontitis. The pooled OR was 1.83 (95%-CI: 1.40, 2.38, *p* < 0.01), and substantial heterogeneity between the studies was found (I^2^ = 77%, *p* < 0.01) (Fig. 3a), indicating that a high TC level is a risk factor for periodontitis. Meta-regression showed that the sources of the heterogeneity were the year of publication (*p* < 0.001), study design (*p* < 0.001), age ratio (*p* < 0.001), P diagnosis (*p* = 0.001) and adjusted OR (*p* = 0.001) (Table S5). The result was robust regardless if any one study was omitted (Supplementary Fig. 2-a). A significant publication bias was found after Egger’s (*p* < 0.001) and Begg’s tests (*p* = 0.021).

ii. TG and periodontitis

Twenty-six studies evaluated the difference in the serum TG level between periodontitis and healthy control groups with the mean (SD). TG levels were higher in periodontitis patients than in controls, with a pooled mean difference of 23.92 mg/dL (95%-CI: 15.52, 32.31, *p* < 0.01). There was significant heterogeneity between the studies (I^2^ = 100%, *p* = 0) (Fig. 2b). The meta-regression did not find any significant source of heterogeneity (Table S5). The result was robust regardless if any one study was omitted (Supplementary Fig. 1-b). No significant publication bias was found after Egger’s(*p* = 0.336) and Begg’s tests (*p* = 0.061).

Seventeen studies reported the OR values to evaluate the association between the serum TG level and periodontitis. The pooled odds ratio was 1.58 (95%-CI: 1.32, 1.90). We used the random effect model due to the presence of heterogeneity between studies (I^2^ = 71%, *p* < 0.01) (Fig. 3b), indicating that TG levels were significantly associated with periodontitis and that high TG levels are a risk factor for periodontitis. Meta-regression showed that the sources of the heterogeneity were the year of publication (*p* = 0.004), study design (*p* < 0.001), total sample size (*p* < 0.001), age (*p* = 0.001), P diagnosis (*p* = 0.001) and adjusted OR (*p* = 0.001) (Table S5). The result was robust regardless of if any one study was omitted (Supplementary Fig. 2-b). A significant publication bias was found after Egger’s(*p* = 0.003) and Begg’s tests(*p* = 0.006) .

iii. LDL and periodontitis

Twenty two studies evaluated the difference in the serum LDL level between periodontitis and healthy control groups with the mean (SD). LDL levels were higher in periodontitis patients than in controls, with a pooled mean difference of 6.26 mg/dL (95% CI: 1.91, 10.61, *p* < 0.01). There was significant heterogeneity between the studies (I^2^ = 92%, *p* < 0.01). (Fig. 2c) However, the meta-regression did not find any significant source of heterogeneity (Table S5). The result was robust regardless if any one study was omitted (Supplementary Fig. 1-c). No significant publication bias was found after Egger’s(*p* = 0.277) and Begg’s tests (*p* = 0.271).

Nine studies reported the OR values to evaluate the association between the serum LDL level and periodontitis. The pooled OR was 2.11 (95%-CI: 1.32, 3.40, *p* < 0.01), indicating that LDL levels were significantly associated with periodontitis and that high LDL levels are a risk factor for periodontitis. We used the random effect model due to the presence of heterogeneity between studies (I^2^ = 71%, *p* < 0.01) (Fig. 3c). Meta-regression showed that the sources of the heterogeneity were the study design (*p* < 0.001), total sample size (*p* = 0.014), and P diagnosis (*p* = 0.018) (Table S5). The result was robust regardless if any one study was omitted (Supplementary Fig. 2-c). A significant publication bias was found after Egger’s(*p* = 0.277) and Begg’s tests (*p* = 0.271).

iv. HDL and periodontitis

Twenty-four studies evaluated the difference in the serum HDL level between periodontitis and healthy control groups with the mean (SD). The HDL levels were lower in periodontitis patients, and the pooled mean difference for the HDL levels in the periodontitis patients and healthy control groups was − 3.45 mg/dL (95%-CI: − 4.47, − 2.44 mg/dL, *p* < 0.01). There was significant heterogeneity between the studies (I^2^ = 64%, *p* < 0.01) (Fig. 2d). Meta-regression showed that age may be the source of the heterogeneity (*p* = 0.003) (Table S5). The result was robust regardless if any one study was omitted (Supplementary Fig. 1-d). No significant publication bias was found after Egger’s(*p* = 0.427) and Begg’s tests (*p* = 0.843).

Thirteen studies reported the OR values to evaluate the association between the serum HDL level and periodontitis. The pooled OR was 1.25 (95% CI: 1.15, 1.35, *p* < 0.01), indicating that a low HDL level is a risk factor for periodontitis. There was no significant evidence for heterogeneity between the studies (I^2^ = 0%, *p* = 0.64) (Fig. 3d). The result was robust regardless if any one study was omitted (Supplementary Fig. 2-d). A significant publication bias was found after Egger’s(*p* = 0.030) and Begg’s tests (*p* = 0.961).

xxii. VLDL and periodontitis

Four studies evaluated the difference in the serum VLDL level between periodontitis and healthy control groups with the mean (SD). The VLDL levels were higher in periodontitis patients than in controls, with a pooled mean difference of 8.69 mg/dL (95% CI: 2.20, 15.18, *p* < 0.01). There was significant heterogeneity between the studies (I^2^ = 81%, *p* < 0.01) (Fig. 2e). No significant publication bias was found after Egger’s(*p* = 0.266). and Begg’s tests (*p* = 0.174).

Some studies were not included in our meta-analysis due to the lack of information utilized. Saxlin T reported an association between high serum triglycerides and low HDL-cholesterol levels with periodontal pockets by quintiles [39]. Akkaloori Anitha stated that the mean LDL and VLDL levels were significantly higher and the HDL levels were lower in periodontal patients than in healthy controls [12].

## Association between dyslipidemia and periodontitis: dyslipidemia as the outcome

### Periodontitis and dyslipidemia

**Fig. 4:**
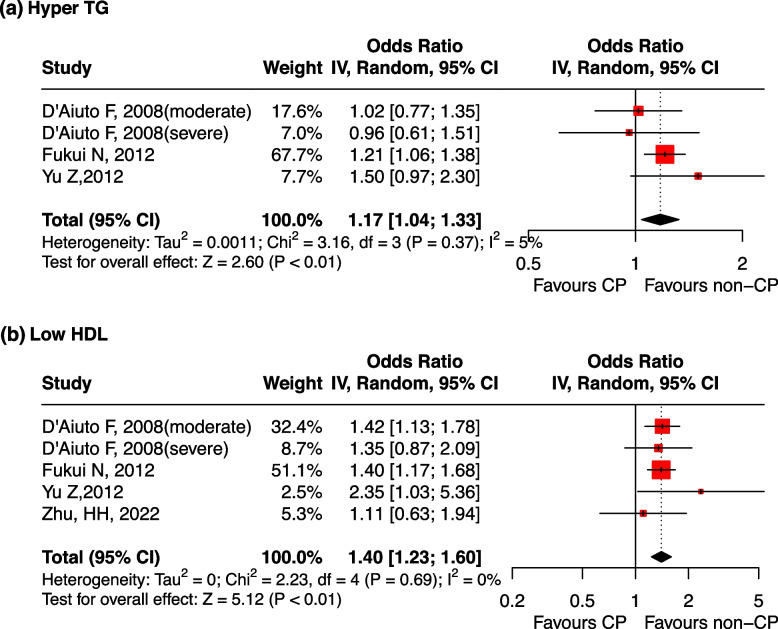
Forest plot of OR values for periodontitis with the risk of dyslipidemia. **a** periodontitis is associated with the high risk of hyper TG; (**b**) periodontitis is associated with the high risk of low HDL. Abbreviations: TG: triglycerides, HDL: high-density lipoprotein, CP: Chronic Periodontitis

Three studies reported OR values to evaluate the association between periodontitis and dyslipidemia. Periodontitis was a risk factor for abnormal increases in TG levels, with a pooled OR of 1.17 (95% CI: 1.04, 1.33). There was no significant heterogeneity between studies (I^2^ = 5%, *p* = 0.37) (Fig. 4a). The result was meaningless when the study by Fukui N, 2012 was omitted (Supplementary Fig. 3-a). No significant publication bias was found after Egger’s(*p* = 0.769) and Begg’s tests (*p* = 1.000).

Periodontitis was a risk factor for abnormal decreases in HDL levels, with a pooled OR of 1.40 (95% CI: 1.23, 1.60, *p* < 0.01), and there was no significant heterogeneity among the studies (I^2^ = 0%, *p* = 0.69) (Fig. 4b). The result was robust regardless if any one study was omitted (Supplementary Fig. 3-b). No significant publication bias was found after Egger’s(*p* = 0.746) and Begg’s tests (*p* = 1.000).

Since the pathological changes in other indicators, including TC, LDL and VLDL, are often not regarded as classic indicators of dyslipidemia, we only analysed the results of hyper TG and low LDL.

i. PD and dyslipidemia

**Fig. 5:**
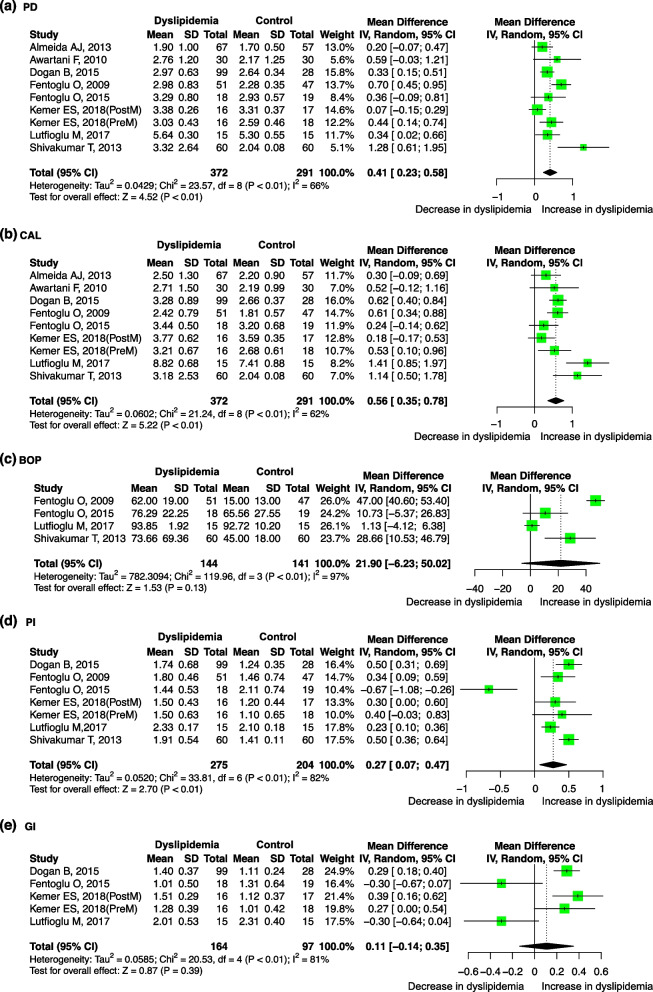
Forest plot of mean difference for comparisons: dyslipidemia versus non- dyslipidemia (**a**) dyslipidemia have deeper PD; (**b**) dyslipidemia have more CAL (**c**) dyslipidemia have more BOP but not significant; (**d**) dyslipidemia have bigger PI; (**e**) dyslipidemia have bigger GI but not significant, Abbreviations: PD: probing depth, CAL: clinical attachment level, BOP: bleeding on probing, PI: plaque index, GI: gingival index

Eight studies evaluated the difference in the PD level between dyslipidemia patients and healthy control groups with the mean (SD). The PD levels were higher in dyslipidemia patients than in controls, with a pooled mean difference of 0.41 mm (95%-CI: 0.23, 0.58, *p* < 0.01). There was significant heterogeneity between the studies (I^2^ = 66%, *p* < 0.01) (Fig. 5a). Meta-regression showed that the sources of the heterogeneity may include the year of publication (*p* = 0.038) and region (*p* = 0.038) (Table S6). The result was robust regardless if any one study was omitted (Supplementary Fig. 4-a). No significant publication bias was found after Egger’s(*p* = 0.178) and Begg’s tests (*p* = 0.095).

ii. CAL and dyslipidemia

Eight studies evaluated the difference in the CAL level between dyslipidemia patients and healthy control groups with the mean (SD). The CAL levels were higher in dyslipidemia patients, with a pooled mean difference of 0.56 mm (95%-CI: 0.35, 0.78, *p* < 0.01). There was significant heterogeneity between studies (I^2^ = 62%; *p* < 0.01) (Fig. 5b). However, no significant source of heterogeneity was found through the meta-regression (Table S6). The result was robust regardless if any one study was omitted (Supplementary Fig. 4-b). No significant publication bias was found after Egger’s (*p* = 0.519) and Begg’s tests (*p* = 0.532).

iii. BOP and dyslipidemia

Four studies evaluated the difference in the BOP level between dyslipidemia patients and healthy control groups with the mean (SD). No significant difference in BOP levels was found between dyslipidemia patients and healthy controls. There was significant heterogeneity between studies (I^2^ = 97%; *p* < 0.01) (Fig. 5c). Meta-regression showed that the sources of the heterogeneity may be the year of publication (*p* < 0.001), total sample size (*p* = 0.004), age (*p* < 0.001) and sex ratio (*p* = 0.002) (Table S6). The result was significant when the study by Lutfioglu M, 2017 [61] was omitted (Supplementary Fig. 4-c). No significant publication bias was found after Egger’s(*p* = 0.848) and Begg’s tests (*p* = 0.497).

iv. PI and dyslipidemia

Six studies evaluated the difference in the PI level between dyslipidemia patients and healthy control groups with the mean (SD). PI levels were higher in dyslipidemia patients, with a pooled mean difference of 0.27 (95%-CI: 0.07, 0.47, *p* < 0.01). There was significant heterogeneity between studies (I^2^ = 82%; *p* < 0.01) (Fig. 5d). Meta-regression showed that the sources of the heterogeneity may be sex (*p* = 0.013) (Table S6). The result was meaningless when the study of Dogan B, 2015 or Shivakumar T, 2013 was omitted (Supplementary Fig. 4-d). No significant publication bias was found after Egger’s(*p* = 0.379) and Begg’s tests (*p* = 0.453).

xxii. GI and dyslipidemia

Four studies evaluated the difference in the GI level between dyslipidemia patients and healthy control groups with a mean (SD). No significant difference in GI level was found between dyslipidemia patients and healthy control groups (Fig. 5e). Meta-regression showed that the sources of the heterogeneity may be Study design(*p* < 0.001), Quality(*p* < 0.001) and Gender ratio(*p* < 0.001) (Table S6). The result was robust regardless if any one study was omitted (Supplementary Fig. 4-e). No significant publication bias was found after Egger’s(*p* = 0.193) and Begg’s tests (*p* = 0.050).

## Effect of periodontal treatment on blood lipids

Three studies evaluated the difference in the association between non-surgical periodontal treatment groups and the control groups with the mean (SD). No significant publication bias was found after Egger’s and Begg’s tests (*p* > 0.05).

i. Nonsurgical periodontal treatment and TC

**Fig. 6:**
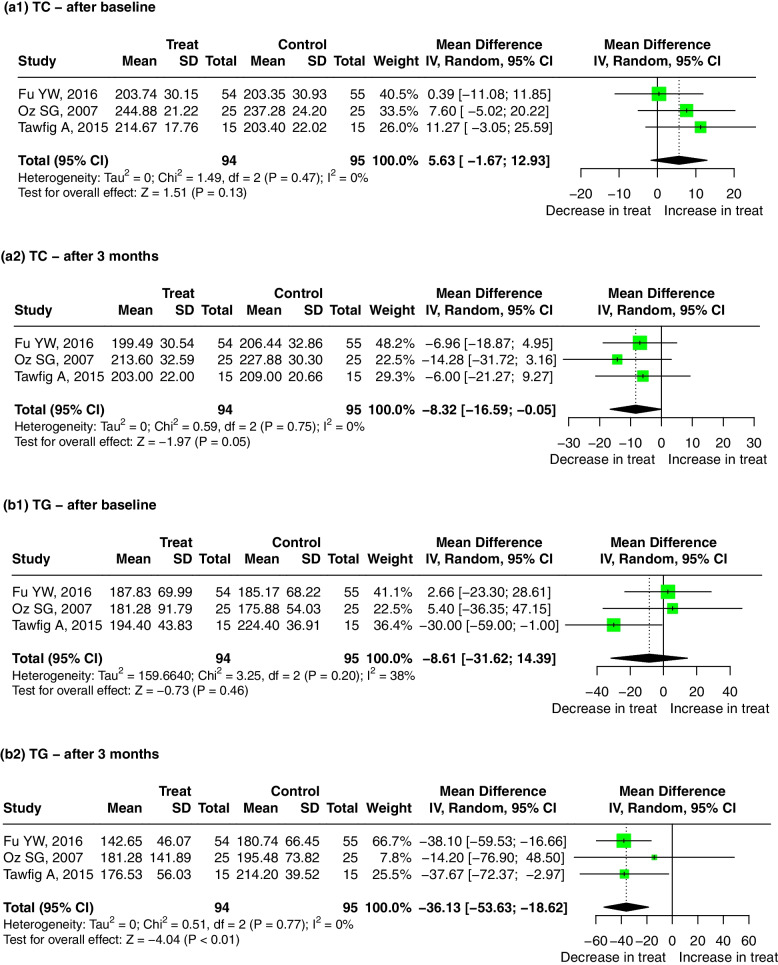

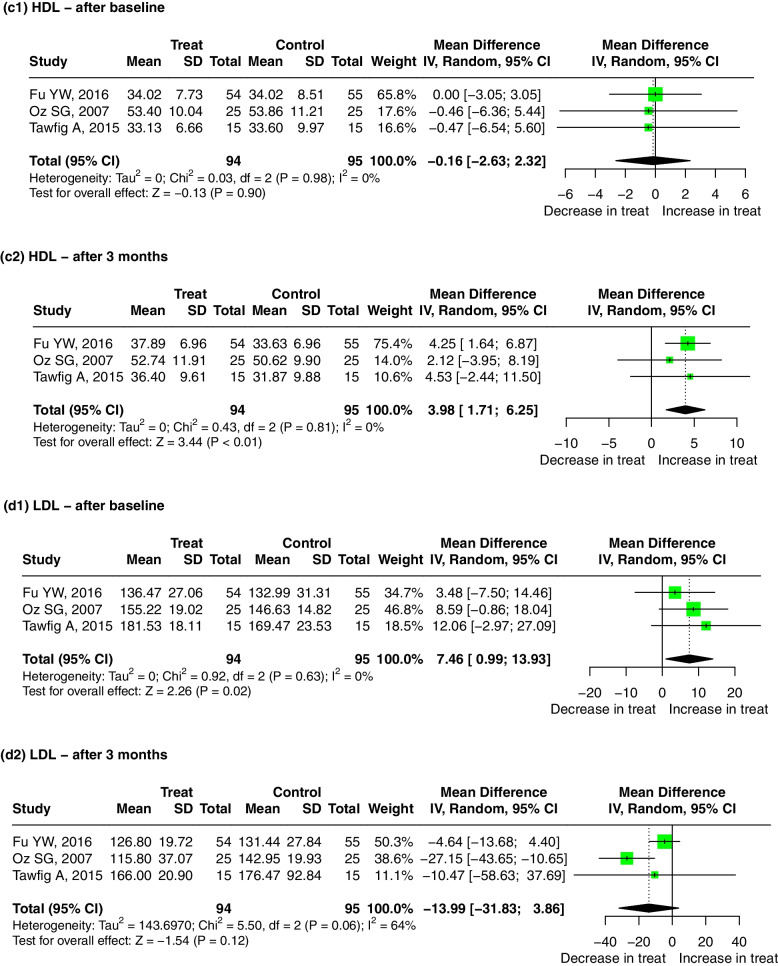
Forest plot of mean difference for comparisons: periodontal treatment versus non-treatment among periodontitis patients. (a1–2) periodontitis treatment can decrease the TC level after 3 months; (b1–2) periodontitis treatment can decrease the TG level after 3 months; (c1–2) periodontitis treatment can increase the HDL level after 3 months; (d1–2) periodontitis treatment do not significantly decrease the LDL level after 3 months. Abbreviations: TC:Total cholesterol, TG: triglycerides, HDL: high-density lipoprotein, LDL: low-density lipoprotein

Compared with the control group, the level of TC in the serum of patients who received a non-surgical periodontal treatment was decreased significantly after 3 months, and the pooled mean difference for TC in the treatment and control groups was − 8.32 mg/dL (95% CI: − 16.59, − 0.05, *p* = 0.05). There was no significant heterogeneity between the studies (I^2^ = 0, *p* = 0.75) (Fig. 6a2). The result was meaningless regardless if any one study was omitted (Supplementary Fig. 5-a2).

Several studies that reported positive results were excluded from the meta-analysis because they did not have the standardized clinical data we needed. DUAN Jinyu et al. reported that 3 months after a nonsurgical periodontal treatment, the cholesterol levels were significantly reduced. With 5.72 mmol/l as the diagnostic criterion, four of eight hypercholesterolemia patients returned to normal serum cholesterol levels [67]. The research by A. Surdumacove produced similar results; compared with the control group that received only oral hygiene guidance, the test group that received a non-surgical periodontal treatment exhibited a significant decrease in TC levels after 1 month [71]. Zuza EP et al. reported an interesting result: after non-surgical periodontal treatments, TC levels in obese patients were significantly reduced 3 months later, but the same results were not observed in nonobese patients [77].

ii. Nonsurgical periodontal treatment and TG

Compared with the control group, the level of TG in the serum of patients who received the non-surgical periodontal treatment was decreased significantly after 3 months, with a pooled mean difference of − 36.13 mmol/L (95% CI: − 53.63, − 18.62, *p* < 0.01). There was no significant heterogeneity between the studies (I^2^ = 0, *p* = 0.77) (Fig. 6b2). The result was robust regardless if any one study was omitted (Supplementary Fig. 5-b2).

Considering the results of other studies, with 1.70 mmol/l as the diagnostic criterion, DUAN Jinyu reported that the serum cholesterol levels in five of 15 hypertriglyceridaemia patients returned to normal after the non-surgical periodontal treatment. The observation period was 3 months [71]. This article was not included in the meta-analysis because there were no specific parameters. Zuza EP also reported similar results [77].

iii. Nonsurgical periodontal treatment and HDL

Compared with the control group, the level of HDL in the serum of patients who received the non-surgical periodontal treatment was increased significantly after 3 months, with a pooled mean difference of 3.98 mmol/L (95% CI: 1.71, 6.25, *p* < 0.01). There was no significant heterogeneity between the studies (I^2^ = 0, *p* = 0.81) (Fig. 6c2). The result was meaningless when the study by Fu YW, 2016 was omitted (Supplementary Fig. 5-c2).

iv. Nonsurgical periodontal treatment and LDL

Finally, we performed a meta-analysis of the LDL levels in serum. Analysis of these studies showed that there was no statistically significant difference in the LDL levels between the treatment and control groups after 3 months of treatment (Fig. 6d2). The result was significant when the study by Fu YW, 2016 was omitted (Supplementary Fig. 5-a).

## Effect of lipid treatment on periodontitis

Five studies evaluated the difference in the association between the lipid treatment and periodontitis with the mean (SD). No significant publication bias was found after Egger’s and Begg’s tests (*p* > 0.05).

**Fig. 7:**
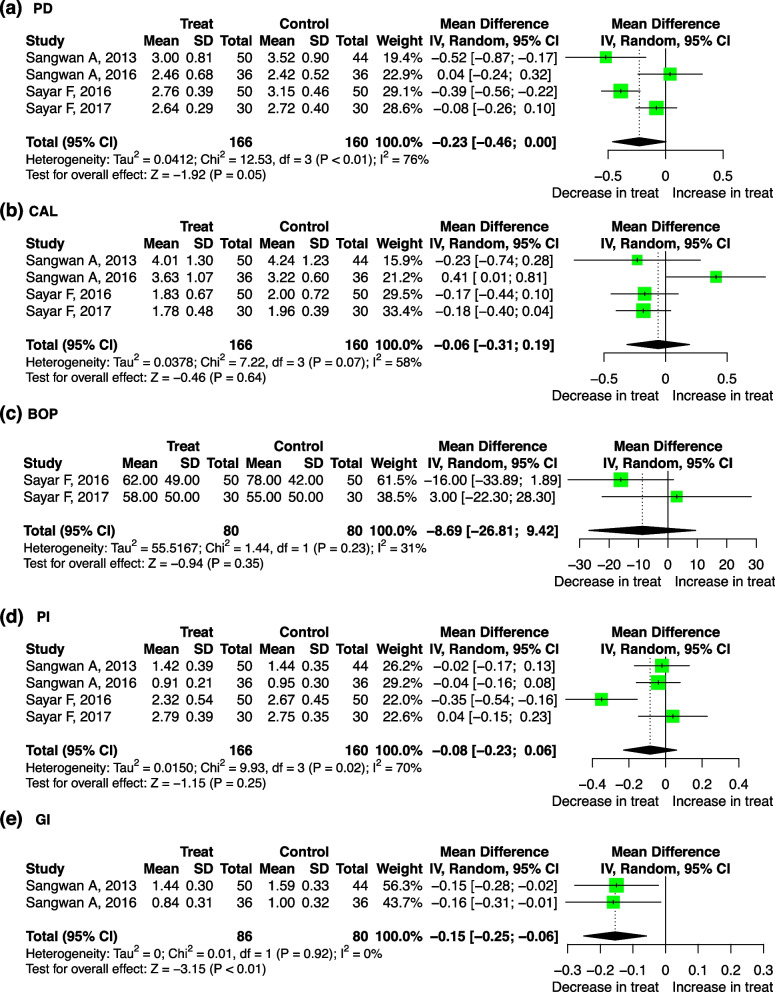
Forest plot of mean difference for comparisons: lipid-lowering treatment versus non-treatment among hyperlipidemia patients. **a** lipid-lowering treatment can decrease PD; (**b**) lipid-lowering treatment do not significantly decrease CAL (**c**) lipid-lowering treatment do not significantly decrease BOP; (**d**) lipid-lowering treatment do not significantly decrease PI; (**e**) lipid-lowering treatment can decrease GI. Abbreviations: PD: probing depth, CAL: clinical attachment level, BOP: bleeding on probing, PI: plaque index, GI: gingival index

Compared with that of the control group, the level of GI in the dyslipidemia patients who received the lipid treatment decreased significantly, with a pooled mean difference of − 0.15 (95%-CI: − 0.25, − 0.06, *p* < 0.01). There was no significant heterogeneity between the studies (I^2^= 0, *p* = 0.92) (Fig. 7e).

We found no statistically significant difference in PD, CAL, BOP, or PI between the treatment and control groups (Fig. 7). Through meta-regression, it was determined that the sources of the heterogeneity may be the total sample size for PD (*p* = 0.017), study design for CAL (*p* = 0.007), and age for PI (*p* = 0.028) (Table S7).

The following results are reported in related studies that are not included in the forest map. Özlem FENTOĞLU reported that 2 months after the periodontal treatment and lipid treatment, PI, GI, BOP, and PD in the statin treatment group were significantly reduced, while similar results were observed in the diet control group [57].

## Discussion

Dyslipidemia is a representative metabolic disease, which is widely speculated to be the important agents that promotes periodontitis. In our study, we set out to determine whether dyslipidemia has similar effects on periodontitis as diabetes mellitus and if it reduces blood lipid levels to help treat periodontitis in patients with dyslipidemia.

Our research results are as follows: first, increasing plasma TC, TG, LDL and reduced HDL levels were risk factors for periodontitis. The periodontal parameters CAL, PD and PI of patients with dyslipidemia were significantly worse. Second, compared with that of the baseline, the plasma lipid levels of patients with dyslipidemia who completed the periodontal treatment were significantly improved after 3 months. Third, for patients with dyslipidemia, periodontal parameters except GI were not significantly improved with statins when compared with the diet control therapy. Our research has identified the association between dyslipidemia and periodontitis, we speculate that cytokines may be the key to linking the two diseases. Bacteria are the major pathogenic factors of periodontal disease. The stimulation of microbes promotes the secretion of cytokines in hosts to promote inflammation by autocrine or paracrine signalling [89]. Bacteria are very important in promoting the progression of periodontitis and the pathological manifestations of active periodontitis. For example, IL-1 and TNF-α affect the function of endothelial cells, leading to the accumulation of neutrophils and monocytes at the site of inflammation [90]. Probing depth (PD), clinical attachment loss (CAL), and bleeding on probing (BOP) are closely related to the increase in MMP levels, pathogens in dental plaque are able to stimulate host cells to increase their MMP release [91]. By means of the analysis of 10 researches, including 485 periodontitis patients and 379 healthy controls, Lin Zhang find that the salivary MMP-8 levels were significantly higher in periodontitis patients compared with healthy controls [92]. Indeed, a decrease in the levels of MMPs in the crevicular fluid has been observed after the treatment of periodontitis. This demonstrates that the levels of MMPs are in a dynamic balance with the state of hygiene and health of periodontal tissues [93, 94]. These inflammatory factors are also related to the development of dyslipidemia.

Several lines of evidence suggested that patients with dyslipidemia exhibited higher TNF-α plasma concentrations, which correlated significantly with the concentrations of VLDL, triglycerides and cholesterol and correlated negatively with HDL cholesterol [93–95]. The use of fenofibrate to treat hyperlipoproteinemia IIB leads to decreased levels of TC, TG, and LDL, which correlate with a decreased concentration of TNF-α [96].

Özlem Fentoğlu found significant correlations between serum and gingival crevicular fluid cytokines (IL-1β and TNF-α) and the TC/HDL ratio in patients with dyslipidemia [56]. A study showed that plasma free fatty acid and glycerol concentrations increased transiently after an injection of TNF [97].

Based on the studies above, we found that cytokines (especially TNF-α) play a critical role in the occurrence and development of periodontitis and dyslipidemia. Perhaps this is an important reason why the two diseases interact with each other. We speculate that the treatment of one disease may also affect the development of the other.

As a standard method for treating periodontitis, non-surgical periodontal treatment has been used in clinical work for a long time. Many studies have shown that after an effective periodontal treatment, the blood lipid levels in plasma are significantly improved. Research by Fu YW et al. showed that the levels of TNF-α, IL-1β, and IL-6 in the periodontal treatment group were significantly lower than those treated only with supragingival scaling [69].

As a conventional drug for the treatment of dyslipidemia, statins have been reported to inhibit the immune reactivity of inflammatory cells [98]. Lin SK found that simvastatin inhibited the effects of TNF-α in a dose-dependent manner [99]. Several studies have documented that when atorvastatin gel is placed subgingivally as an adjunct to scaling and root planning, it leads to significant periodontal regeneration [100, 101]. However, in our study, one unanticipated result was that for patients with dyslipidemia who received the systemic therapy, statins did not significantly improve periodontal parameters except GI when compared with that of the diet control therapy. The limited number of studies available may undermine the accuracy of the results.

This study indicates that there is a bi-directional correlation between dyslipidemia and chronic periodontitis. Controlling blood lipid levels may improve the effect of non-surgical periodontal treatments on periodontitis. Maintaining periodontal health is also beneficial for the conditions of lipids in dyslipidemia patients. We can also perform combined treatment when necessary.

We are aware that our study has limitations that should be considered. First, studies with invalid or negative results tend not to be published, so it is difficult to completely prevent publication bias. Second, due to the different diagnoses of periodontitis or dyslipidemia in different countries, our inclusion criteria cannot be completely unified. In this study, significant heterogeneity was found, perhaps due to the region (European/Americas or Asian), criterion of Pd diagnosis, publication year, study design, age ratio, etc., which may undermine the validity of the results. Third, regarding the effect of lipid treatments on periodontitis, the limited number of available studies limits the ability to obtain a comprehensive result.

## Conclusion

Overall, we can conclude that there is a bi-direction relationship between dyslipidemia and periodontitis. Periodontal therapy can improve the condition of dyslipidemia, but we did not observe a periodontitis-improving effect when statins were systematically used.

### Supplementary Information

**Additional file 1.**

**Additional file 2.**

**Additional file 3.**

**Additional file 4.**

**Additional file 5.**

**Additional file 6.**

**Additional file 7.**

**Additional file 8.**

**Additional file 9.**

**Additional file 10.**

**Additional file 11.**

**Additional file 12.**

**Additional file 13.**

## Data Availability

All data generated or analyzed during the present study are included in this published article.

## Abbreviations

TC: Total Cholesterol
TGs: Triglycerides
LDL: Low-density lipoprotein
HDL: High-density lipoprotein
GI: Gingival index
Pd: Periodontal disease
T2DM: Type 2 diabetes mellitus
IBD: Inflammatory bowel disease
PD: Probing Depth
CAL: Clinical attachment level
BOP: Bleeding on probing
PI: Plaque index
CVD: Cardiovascular disease
CPI: Community periodontal index
NOS: Newcastle–Ottawa Scale
AHRQ: Agency for Healthcare Research and Quality
MD: Mean difference
CI: Confidence interval
