# Supplementary material for: Exploring the bi-directional relationship between periodontitis and dyslipidemia: a comprehensive systematic review and meta-analysis
Source: BMC Oral Health. 2024 Apr 29;24:508. doi: 10.1186/s12903-023-03668-7 (PMC11059608; doi:10.1186/s12903-023-03668-7)
Supplement: Supplementary file 1 — Additional file 1. [file 12903_2023_3668_MOESM1_ESM.docx]

Table S1. Quality assessment for case-control studies with Newcastle - Ottawa Scale (NOS)

| Study | Q1 | Q2 | Q3 | Q4 | Q5 | Q6 | Q7 | Q8 | n (%) of stars / quality |
| --- | --- | --- | --- | --- | --- | --- | --- | --- | --- |
| Cury EZ, 2018 | * | * |  | * | * | * | * |  | 6 (66.7%) / Moderate |
| Cutler CW, 1999 | * |  |  | * | * | * | * |  | 5 (55.6%) / Moderate |
| Doraiswamy S, 2017 | * | * |  | * | * | * | * |  | 6 (66.7%) / Moderate |
| Fentoglu O, 2009 | * | * |  | * | ** | * | * |  | 7 (77.8%) / High |
| Fentoglu O, 2011 | * | * |  | * | * | * | * |  | 6 (66.7%) / Moderate |
| Fentoglu O, 2015 | * | * |  | * | * | * | * |  | 6 (66.7%) / Moderate |
| Fentoglu O, 2020 | * | * |  | * | ** | * | * |  | 7 (77.8%) / High |
| Gao H, 2015 | * | * |  | * | * | * | * |  | 6 (66.7%) / Moderate |
| Golpasand HL, 2014 | * | * |  | * | * | * | * |  | 6 (66.7%) / Moderate |
| Hamissi J, 2011 | * |  |  | * | * | * | * |  | 5 (55.6%) / Moderate |
| Kalburgi V, 2014 | * |  |  | * | * | * | * |  | 5 (55.6%) / Moderate |
| Losche W, 2000 | * | * |  | * | * | * | * |  | 6 (66.7%) / Moderate |
| Lutfioglu M,2017 | * | * |  | * | * | * | * |  | 6 (66.7%) / Moderate |
| Machado AC, 2005 | * |  |  | * | * | * | * |  | 5 (55.6%) / Moderate |
| Moeintaghavi A, 2005 | * | * |  | * | * | * | * |  | 6 (66.7%) / Moderate |
| Moghadam SA, 2015 | * | * |  | * | * | * | * |  | 6 (66.7%) / Moderate |
| Monteiro AM, 2009 | * | * |  | * | ** | * | * |  | 7 (77.8%) / High |
| Nibali L, 2007 | * | * |  | * | * | * | * |  | 6 (66.7%) / Moderate |
| Penumarthy S, 2013 | * |  |  | * |  | * | * |  | 4 (44.4%) / Low |
| Scardina GA, 2011 | * |  |  | * |  | * | * |  | 4 (44.4%) / Low |
| Shi D, 2006 | * |  |  | * | * | * | * |  | 5 (55.6%) / Moderate |
| Shivakumar T, 2013 | * |  |  | * |  | * | * |  | 4 (44.4%) / Low |
| Sridhar R, 2009 | * |  |  | * |  | * | * |  | 4 (44.4%) / Low |

*Note:* NOS for case-control studies: (Q1) Is the case definition adequate? (Q2) Representativeness of the cases (Q3) Selection of Controls (Q4) Definition of Controls (Q5) Comparability of cases and controls on the basis of the design or analysis (Q6) Ascertainment of exposure (Q7) Same method of ascertainment for cases and controls (Q8) Non-Response rate. A total of eight stars.
