# Supplementary material for: Exploring the bi-directional relationship between periodontitis and dyslipidemia: a comprehensive systematic review and meta-analysis
Source: BMC Oral Health. 2024 Apr 29;24:508. doi: 10.1186/s12903-023-03668-7 (PMC11059608; doi:10.1186/s12903-023-03668-7)
Supplement: Supplementary file 2 — Additional file 2. [file 12903_2023_3668_MOESM2_ESM.docx]

Table S2. Quality assessment for cohort studies with Newcastle - Ottawa Scale (NOS)

| Study | Q1 | Q2 | Q3 | Q4 | Q5 | Q6 | Q7 | Q8 | n (%) of stars / quality |
| --- | --- | --- | --- | --- | --- | --- | --- | --- | --- |
| Duan JY, 2009 | * | * | * | * | * | * | * |  | 7 (77.8%) / High |
| Fentoglu O, 2010 | * | * | * | * | * | * | * |  | 7 (77.8%) / High |
| Fentoglu O, 2015 |  | * | * | * | * | * | * |  | 6 (66.7%) / Moderate |
| Losche W, 2005 |  |  | * | * | * | * | * |  | 5 (55.6%) / Moderate |
| Macovei-Surdu A, 2013 |  | * | * | * | * | * | * |  | 6 (66.7%) / Moderate |
| Nibali L, 2015 | * |  | * | * | ** | * | * |  | 7 (77.8%) / High |
| Nicolaiciuc O, 2016 |  |  | * | * | * | * | * |  | 5 (55.6%) / Moderate |
| Sangwan A, 2016 | * | * | * | * | ** | * | * |  | 8 (88.9%) / High |
| Taleghani F, 2010 | * |  | * | * | ** | * |  |  | 6 (66.7%) / Moderate |
| Zuza EP, 2016 | * | * | * | * | * | * | * |  | 7 (77.8%) / High |

*Note:* NOS for cohort studies: (Q1) Representativeness of the exposed cohort (Q2) Selection of the non exposed cohort (Q3) Ascertainment of exposure (Q4) Demonstration that outcome of interest was not present at start of study (Q5) Comparability of cohorts on the basis of the design or analysis (Q6) Assessment of outcome (Q7) Was follow-up long enough for outcomes to occur (Q8) Adequacy of follow up of cohorts. A total of eight stars.
