# Supplementary material for: Exploring the bi-directional relationship between periodontitis and dyslipidemia: a comprehensive systematic review and meta-analysis
Source: BMC Oral Health. 2024 Apr 29;24:508. doi: 10.1186/s12903-023-03668-7 (PMC11059608; doi:10.1186/s12903-023-03668-7)
Supplement: Supplementary file 3 — Additional file 3. [file 12903_2023_3668_MOESM3_ESM.docx]

Table S3. Quality assessment for cross-sectional studies with Healthcare Research and Quality (AHRQ) checklist

| Study | Q1 | Q2 | Q3 | Q4 | Q5 | Q6 | Q7 | Q8 | Q9 | Q10 | Q11 | n (%) of Y / quality |
| --- | --- | --- | --- | --- | --- | --- | --- | --- | --- | --- | --- | --- |
| Almeida AJ, 2013 | Y | Y | Y | Y | N | Y | Y | Y | N | N | *NA* | 7 (63.6%) / Moderate |
| Al-Otaibi DH, 2008 | Y | Y | Y | U | N | N | Y | N | N | N | *NA* | 4 (36.4%) / Low |
| Anitha A, 2014 | Y | Y | Y | Y | N | N | Y | Y | N | N | *NA* | 6 (54.5%) / Moderate |
| Awartani F, 2010 | Y | Y | Y | Y | N | Y | Y | N | N | N | *NA* | 6 (54.5%) / Moderate |
| Banihashemrad SA, 2008 | Y | N | N | U | N | N | N | N | N | N | *NA* | 1 (9.1%) / Low |
| Bullon P, 2014 | Y | Y | Y | Y | N | N | Y | N | N | Y | *NA* | 6 (54.5%) / Moderate |
| Chen CC, 2021 | Y | Y | Y | Y | N | Y | N | N | N | N | NA | 5 (45.5%) / Moderate |
| D'Aiuto F, 2008 | Y | Y | Y | Y | N | Y | Y | Y | N | N | *NA* | 7 (63.6%) / Moderate |
| Ding CY, 2023 | Y | Y | Y | Y | N | Y | N | Y | N | N | NA | 5 (45.5%) / Moderate |
| Dogan B, 2015 | Y | Y | Y | Y | N | Y | Y | Y | N | N | *NA* | 7 (63.6%) / Moderate |
| Fentoglu O, 2011 | Y | Y | Y | Y | N | N | Y | Y | N | N | *NA* | 6 (54.5%) / Moderate |
| Fukui N, 2012 | Y | Y | Y | Y | N | Y | Y | Y | N | N | *NA* | 7 (63.6%) / Moderate |
| Güler B, 2020 | Y | Y | Y | Y | N | N | Y | N | N | N | *NA* | 5 (45.5%) / Moderate |
| Guan XY, 2022 | Y | Y | Y | Y | N | N | Y | N | N | N | NA | 5 (45.5%) / Moderate |
| Han SJ, 2019 | Y | Y | Y | Y | N | N | Y | Y | N | N | *NA* | 6 (54.5%) / Moderate |
| Katz J, 2002 | Y | Y | Y | Y | N | Y | Y | Y | N | N | *NA* | 7 (63.6%) / Moderate |
| Kemer ES, 2018 | Y | Y | Y | Y | N | Y | Y | Y | N | N | *NA* | 7 (63.6%) / Moderate |
| Kim SR, 2020 | Y | Y | Y | Y | N | N | Y | Y | N | N | *NA* | 6 (54.5%) / Moderate |
| Koshy BS, 2017 | Y | Y | Y | Y | N | N | Y | N | N | N | *NA* | 5 (45.5%) / Moderate |
| Kumar KR, 2014 | Y | Y | N | N | N | Y | N | N | N | N | *NA* | 3 (27.3%) / Low |
| Kushiyama M, 2009 | Y | Y | Y | Y | N | N | Y | Y | N | Y | *NA* | 7 (63.6%) / Moderate |
| Lee JB, 2013 | Y | Y | Y | Y | N | N | Y | Y | N | N | *NA* | 6 (54.5%) / Moderate |
| Lee S, 2018 | Y | Y | Y | Y | N | Y | Y | Y | N | N | *NA* | 7 (63.6%) / Moderate |
| Sandi RM, 2014 | Y | Y | Y | Y | N | Y | N | N | N | N | *NA* | 5 (45.5%) / Moderate |
| Sangwan A, 2013 | Y | Y | Y | Y | Y | Y | Y | Y | N | N | *NA* | 8 (72.7%) / High |
| Saxlin T, 2008 | Y | Y | Y | Y | N | Y | Y | Y | N | Y | *NA* | 8 (72.7%) / High |
| Sayar F, 2016 | Y | Y | Y | Y | Y | Y | Y | Y | N | N | *NA* | 8 (72.7%) / High |
| Sayar F, 2017 | Y | Y | Y | Y | Y | Y | Y | Y | N | N | *NA* | 8 (72.7%) / High |
| Shimazaki Y, 2007 | Y | Y | Y | Y | N | Y | Y | Y | N | N | *NA* | 7 (63.6%) / Moderate |
| Thapa S, 2016 | Y | Y | Y | Y | N | N | Y | Y | N | Y | *NA* | 7 (63.6%) / Moderate |
| Thomas B, 2017 | Y | Y | Y | Y | N | N | Y | N | N | N | *NA* | 5 (45.5%) / Moderate |
| Wang Y, 2007 | Y | Y | Y | Y | N | Y | Y | Y | N | N | *NA* | 7 (63.6%) / Moderate |
| Yu Z，2012 | Y | Y | Y | Y | N | Y | Y | Y | N | N | *NA* | 7 (63.6%) / Moderate |
| Zhang F, 2022 | Y | Y | Y | N | N | N | Y | Y | N | N | NA | 5 (45.5%) / Moderate |
| Zhou SY, 2012 | Y | Y | N | Y | N | Y | Y | Y | N | N | *NA* | 6 (54.5%) / Moderate |
| Zhu HH, 2022 | Y | Y | Y | Y | N | Y | Y | Y | N | N | NA | 7 (63.6%) / Moderate |

*Note:* AHRQ Checklist for Cross Sectional Studies: (Q1) Define the source of information (survey, record review) (Q2) List inclusion and exclusion criteria for exposed and unexposed subjects (cases and controls) or refer to previous publications (Q3) Indicate time period used for identifying patients (Q4) Indicate whether or not subjects were consecutive if not population-based (Q5) Indicate if evaluators of subjective components of study were masked to other aspects of the status of the participants (Q6) Describe any assessments undertaken for quality assurance purposes (e.g., test/retest of primary outcome measurements) (Q7) Explain any patient exclusions from analysis (Q8) Describe how confounding was assessed and/or controlled (Q9) If applicable, explain how missing data were handled in the analysis (Q10) Summarize patient response rates and completeness of data collection. (Q11) Clarify what follow-up, if any, was expected and the percentage of patients for which incomplete data or follow-up was obtained

Abbreviations: Y, yes; N, no; U, unclear; *NA*: not applicable
