# Supplementary material for: Exploring the bi-directional relationship between periodontitis and dyslipidemia: a comprehensive systematic review and meta-analysis
Source: BMC Oral Health. 2024 Apr 29;24:508. doi: 10.1186/s12903-023-03668-7 (PMC11059608; doi:10.1186/s12903-023-03668-7)
Supplement: Supplementary file 4 — Additional file 4. [file 12903_2023_3668_MOESM4_ESM.docx]

Table S4. Cochrane Collaboration’s tool for assessing risk of bias of randomised clinical trials

| Study | Q1 | Q2 | Q3 | Q4 | Q5 | Q6 | Q7 | Risk of bias |
| --- | --- | --- | --- | --- | --- | --- | --- | --- |
| Fu YW, 2016 | L | L | H | H | L | U | U | High risk |
| Nassar PO, 2011 | U | U | U | U | U | U | U | Unclear risk |
| Oz SG, 2007 | U | U | U | L | U | U | U | Unclear risk |
| Tawfig A, 2015 | U | U | U | U | U | U | U | Unclear risk |

*Note:* Cochrane Collaboration’s tool for assessing risk of bias of randomised clinical trials, source of bias: (Q1) Random sequence generation (Q2) Allocation concealment (Q3) Blinding of participants and personnel (Q4) Blinding of outcome assessment (Q5) Incomplete outcome data (Q6) Selective reporting (Q7) Anything else, ideally prespecified

Abbreviations: H, high; L, Low; U, unclear
