# Supplementary material for: Exploring the bi-directional relationship between periodontitis and dyslipidemia: a comprehensive systematic review and meta-analysis
Source: BMC Oral Health. 2024 Apr 29;24:508. doi: 10.1186/s12903-023-03668-7 (PMC11059608; doi:10.1186/s12903-023-03668-7)
Supplement: Supplementary file 5 — Additional file 5. [file 12903_2023_3668_MOESM5_ESM.docx]

Table S5. Meta-regression analysis of covariates as potential sources of heterogeneity for the association between dyslipidemia and periodontitis：periodontitis as the outcome

| Variables | TC | | |  | TG | | |  | LDL | | |  | HDL | | |
| --- | --- | --- | --- | --- | --- | --- | --- | --- | --- | --- | --- | --- | --- | --- | --- |
|  | beta | SE | P - value |  | beta | SE | P - value |  | beta | SE | P - value |  | beta | SE | P - value |
| **Effect index: MD** |  |  |  |  |  |  |  |  |  |  |  |  |  |  |  |
| Year of publication | -0.61 | 0.43 | 0.156 |  | -1.53 | 0.81 | 0.057 |  | -0.35 | 0.44 | 0.419 |  | -0.07 | 0.10 | 0.519 |
| Region (European/Americas vs. Asian) | -0.10 | 8.23 | 0.991 |  | 6.90 | 11.52 | 0.550 |  | 7.85 | 5.63 | 0.163 |  | -0.12 | 1.60 | 0.939 |
| Study design (Cross-sectional vs. others) | -15.14 | 6.99 | 0.030 |  | -18.33 | 8.13 | 0.024 |  | -8.15 | 5.30 | 0.124 |  | 0.03 | 1.31 | 0.982 |
| Total sample size (log10) | -7.84 | 5.99 | 0.191 |  | -5.01 | 5.62 | 0.373 |  | -4.06 | 4.49 | 0.366 |  | 0.06 | 0.97 | 0.951 |
| Quality (percentage) | -0.01 | 0.30 | 0.967 |  | 0.06 | 0.40 | 0.876 |  | -0.08 | 0.22 | 0.731 |  | 0.08 | 0.05 | 0.095 |
| Age (mean) | -0.42 | 0.26 | 0.112 |  | -0.85 | 0.54 | 0.117 |  | -0.11 | 0.23 | 0.629 |  | 0.07 | 0.06 | 0.182 |
| Age age (P/ HC) | 18.86 | 26.72 | 0.480 |  | 16.49 | 31.26 | 0.598 |  | 46.47 | 27.55 | 0.092 |  | -14.96 | 4.66 | 0.001 |
| Gender (% male) | -0.06 | 0.18 | 0.715 |  | 0.142 | 0.319 | 0.656 |  | -0.06 | 0.15 | 0.668 |  | -0.04 | 0.04 | 0.307 |
| Gender ratio (P/ HC) | -17.76 | 16.15 | 0.272 |  | -6.56 | 19.05 | 0.731 |  | -13.15 | 10.95 | 0.230 |  | 0.42 | 2.35 | 0.859 |
| BMI matching | -14.33 | 8.00 | 0.073 |  | -3.42 | 15.67 | 0.827 |  | -5.57 | 7.37 | 0.450 |  | -2.39 | 1.72 | 0.165 |
| P diagnosis | 14.30 | 6.99 | 0.041 |  | 1.48 | 7.64 | 0.847 |  | 5.55 | 5.73 | 0.333 |  | -1.17 | 1.39 | 0.400 |
| **Effect index: OR** |  |  |  |  |  |  |  |  |  |  |  |  |  |  |  |
| Year of publication | -0.07 | 0.02 | <0.001 |  | -0.05 | 0.02 | 0.004 |  | -0.01 | 0.04 | 0.754 |  |  |  |  |
| Region (European/Americas vs. Asian) | 0.15 | 0.31 | 0.628 |  | 0.10 | 0.26 | 0.701 |  | 0.35 | 0.60 | 0.559 |  |  |  |  |
| Study design (Cross-sectional vs. others) | -1.00 | 0.24 | <0.001 |  | -1.14 | 0.22 | <0.001 |  | -1.34 | 0.36 | <0.001 |  |  |  |  |
| Total sample size (log10) | -0.57 | 0.11 | <0.001 |  | -0.46 | 0.10 | <0.001 |  | -0.66 | 0.27 | 0.014 |  |  |  |  |
| Quality (percentage) | 0.01 | 0.02 | 0.542 |  | -0.01 | 0.01 | 0.662 |  | -0.03 | 0.05 | 0.463 |  |  |  |  |
| Age (mean) | -0.03 | 0.03 | 0.379 |  | -0.02 | 0.03 | 0.433 |  | 0.05 | 0.07 | 0.501 |  |  |  |  |
| Age age (P/ HC) | -3.64 | 1.11 | 0.001 |  | -3.86 | 0.91 | <0.001 |  | -3.42 | 3.06 | 0.264 |  |  |  |  |
| Gender (% male) | 0.00 | 0.01 | 0.538 |  | 0.00 | 0.00 | 0.759 |  | -0.01 | 0.01 | 0.354 |  |  |  |  |
| Gender ratio (P/ HC) | -0.16 | 0.85 | 0.849 |  | 0.43 | 0.95 | 0.654 |  | -0.30 | 1.49 | 0.841 |  |  |  |  |
| BMI matching | 0.22 | 0.62 | 0.727 |  | 0.81 | 0.59 | 0.167 |  | 0.57 | 0.94 | 0.546 |  |  |  |  |
| P diagnosis | 0.88 | 0.26 | 0.001 |  | 0.39 | 0.23 | 0.097 |  | 1.37 | 0.58 | 0.018 |  |  |  |  |
| Adjust OR | -0.88 | 0.25 | 0.001 |  | -1.03 | 0.23 | <0.001 |  | -1.34 | 0.36 | <0.001 |  |  |  |  |

Abbreviation: MD, mean difference; OR odds ratio; P, periodontitis; HC, health control; TC, total cholesterol; TG, triglyceride; LDL, low density lipoprotein cholesterol; HDL, high density lipoprotein cholesterol
