# Supplementary material for: Exploring the bi-directional relationship between periodontitis and dyslipidemia: a comprehensive systematic review and meta-analysis
Source: BMC Oral Health. 2024 Apr 29;24:508. doi: 10.1186/s12903-023-03668-7 (PMC11059608; doi:10.1186/s12903-023-03668-7)
Supplement: Supplementary file 6 — Additional file 6. [file 12903_2023_3668_MOESM6_ESM.docx]

Table S6. Meta-regression analysis of covariates as potential sources of heterogeneity for the association between dyslipidemia and periodontitis：dyslipidemia as the outcome (Effect index: mean difference)

| Variables | PD | | |  | CAL | | |  | BOP | | |  | PI | | |  | GI | | |
| --- | --- | --- | --- | --- | --- | --- | --- | --- | --- | --- | --- | --- | --- | --- | --- | --- | --- | --- | --- |
|  | beta | SE | P - value |  | beta | SE | P - value |  | beta | SE | P - value |  | beta | SE | P - value |  | beta | SE | P - value |
| Year of publication | -0.05 | 0.02 | 0.038 |  | -0.01 | 0.04 | 0.882 |  | -5.75 | 0.53 | <0.001 |  | -0.01 | 0.04 | 0.751 |  | 0.08 | 0.12 | 0.508 |
| Region (European/Americas vs. Asian) | -0.57 | 0.28 | 0.038 |  | -0.31 | 0.31 | 0.323 |  | -8.88 | 35.57 | 0.803 |  | -0.28 | 0.29 | 0.326 |  |  |  |  |
| Study design (cross-sectional vs. case-control) | -0.31 | 0.16 | 0.050 |  | -0.33 | 0.23 | 0.151 |  |  |  |  |  | 0.24 | 0.22 | 0.283 |  | 0.61 | 0.14 | <0.001 |
| Total sample size (log10) | 0.35 | 0.35 | 0.320 |  | 0.10 | 0.44 | 0.817 |  | 65.76 | 22.61 | 0.004 |  | 0.59 | 0.34 | 0.084 |  | 0.44 | 0.65 | 0.501 |
| Quality (percentage) | -0.02 | 0.01 | 0.148 |  | -0.02 | 0.01 | 0.217 |  | 0.26 | 1.19 | 0.829 |  | -0.01 | 0.01 | 0.615 |  | 0.18 | 0.04 | <0.001 |
| Age (mean) | 0.04 | 0.05 | 0.441 |  | -0.04 | 0.07 | 0.548 |  | 6.22 | 1.27 | <0.001 |  | 0.08 | 0.07 | 0.209 |  |  |  |  |
| Age ratio (DLP : HC) | -1.96 | 1.74 | 0.260 |  | 3.29 | 2.45 | 0.179 |  | -119.15 | 165.01 | 0.470 |  | 2.36 | 3.06 | 0.440 |  |  |  |  |
| Gender (% male) | 0.01 | 0.00 | 0.217 |  | 0.01 | 0.01 | 0.144 |  | -7.29 | 4.56 | 0.110 |  | 0.00 | 0.01 | 0.452 |  | -0.01 | 0.01 | 0.088 |
| Gender ratio (DLP : HC) | -0.05 | 0.45 | 0.903 |  | 0.32 | 0.56 | 0.563 |  | -73.24 | 23.53 | 0.002 |  | -1.27 | 0.51 | 0.013 |  | -1.34 | 0.32 | <0.001 |

Abbreviation: DLP, dyslipidemia; HC, health control; PD, probing depth; CAL, clinical attachment loss; BOP, bleeding on probing; PI, plaque index; GI, gingival index
