# Supplementary material for: Exploring the bi-directional relationship between periodontitis and dyslipidemia: a comprehensive systematic review and meta-analysis
Source: BMC Oral Health. 2024 Apr 29;24:508. doi: 10.1186/s12903-023-03668-7 (PMC11059608; doi:10.1186/s12903-023-03668-7)
Supplement: Supplementary file 7 — Additional file 7. [file 12903_2023_3668_MOESM7_ESM.docx]

Table S7. Meta-regression analysis of covariates as potential sources of heterogeneity for the dyslipidemia treatment and periodontitis

| Variables | PD | | |  | CAL | | |  | PI | | |
| --- | --- | --- | --- | --- | --- | --- | --- | --- | --- | --- | --- |
|  | beta | SE | P - value |  | beta | SE | P - value |  | beta | SE | P - value |
| Year of publication | 0.11 | 0.08 | 0.161 |  | 0.03 | 0.11 | 0.791 |  | -0.01 | 0.06 | 0.871 |
| Region (Iran vs. Indian) | -0.01 | 0.28 | 0.960 |  | -0.32 | 0.24 | 0.174 |  | -0.12 | 0.16 | 0.452 |
| Study design (cross-sectional vs. cohort) | -0.35 | 0.27 | 0.204 |  | -0.59 | 0.22 | 0.007 |  | -0.07 | 0.22 | 0.761 |
| Total sample size | -0.01 | 0.00 | 0.018 |  | 0.00 | 0.01 | 0.679 |  | -0.01 | 0.00 | 0.143 |
| Age (mean) | -0.06 | 0.08 | 0.460 |  | -0.09 | 0.10 | 0.366 |  | -0.08 | 0.04 | 0.028 |
| Age ratio (treat : control) | -0.44 | 3.96 | 0.911 |  | 1.30 | 4.40 | 0.767 |  | 2.85 | 1.83 | 0.118 |
| Gender (% male) | 0.02 | 0.03 | 0.473 |  | 0.04 | 0.03 | 0.158 |  | 0.02 | 0.02 | 0.144 |
| Gender ratio (treat : control) | 0.95 | 1.16 | 0.415 |  | 0.33 | 1.38 | 0.808 |  | -0.51 | 0.70 | 0.468 |

Abbreviation: PD, probing depth; CAL, clinical attachment loss; PI, plaque index
