# Supplementary material for: Exploring the bi-directional relationship between periodontitis and dyslipidemia: a comprehensive systematic review and meta-analysis
Source: BMC Oral Health. 2024 Apr 29;24:508. doi: 10.1186/s12903-023-03668-7 (PMC11059608; doi:10.1186/s12903-023-03668-7)
Supplement: Supplementary file 8 — Additional file 8. [file 12903_2023_3668_MOESM8_ESM.pdf]

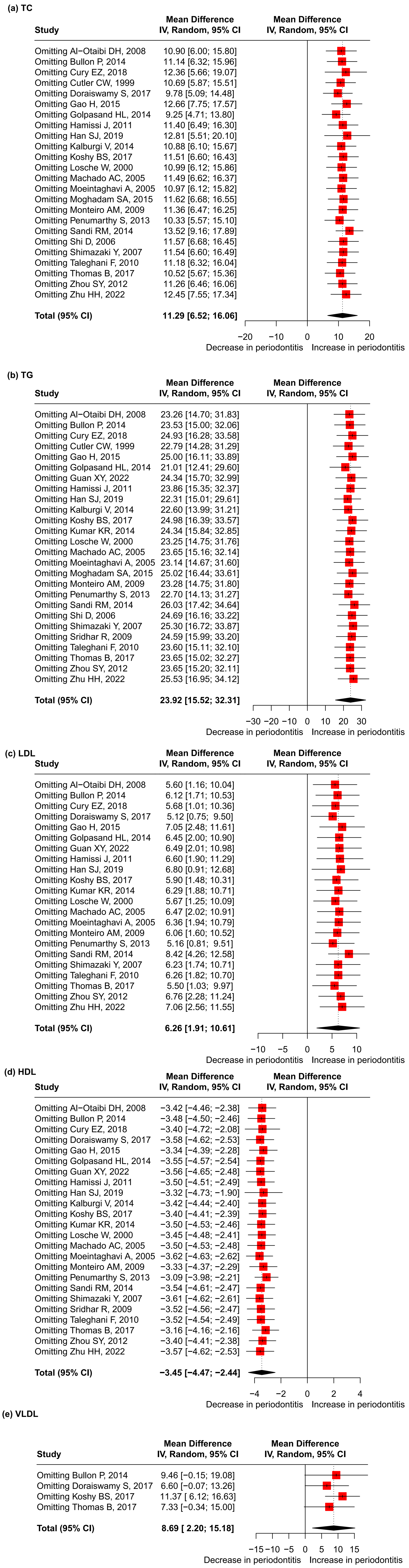

Supplementary Figure 1. Sensitivity analysis of mean difference of lipids levels for comparisons: periodontitis versus non-periodontitis. (a) TC; (b) TG; (c) LDL; (d) HDL; (e) VLDL. Sensitivity analyses were conducted using the leave-one-out method, which removes one study each time and repeats the analysis. The results were robust regardless if any one study was omitted except for VLDL comparison.TC: Total cholesterol, TG: triglycerides, LDL: low-density lipoprotein, HDL: high-density lipoprotein, VLDL: very low density lipoprotein
