# Supplementary material for: Exploring the bi-directional relationship between periodontitis and dyslipidemia: a comprehensive systematic review and meta-analysis
Source: BMC Oral Health. 2024 Apr 29;24:508. doi: 10.1186/s12903-023-03668-7 (PMC11059608; doi:10.1186/s12903-023-03668-7)
Supplement: Supplementary file 9 — Additional file 9. [file 12903_2023_3668_MOESM9_ESM.pdf]

(a) TC

Study	Odds Ratio IV, Random, 95% CI
Omitting Cury EZ, 2018	1.88 [1.43; 2.47]
Omitting Cutler CW, 1999	1.73 [1.34; 2.24]
Omitting Doraiswamy S, 2017	1.66 [1.30; 2.11]
Omitting Golpasand HL, 2014	1.74 [1.34; 2.26]
Omitting Hamissi J, 2011	1.79 [1.37; 2.34]
Omitting Han SJ, 2019	2.00 [1.50; 2.68]
Omitting Kim SR, 2020	2.06 [1.50; 2.84]
Omitting Lee JB, 2013	2.07 [1.49; 2.86]
Omitting Lee S,2018 (Men)	2.00 [1.51; 2.64]
Omitting Lee S,2018 (Women)	1.93 [1.45; 2.56]
Omitting Losche W, 2000	1.79 [1.37; 2.34]
Omitting Machado AC, 2005	1.84 [1.40; 2.40]
Omitting Moeintaghavi A, 2005	1.74 [1.34; 2.25]
Omitting Monteiro AM, 2009	1.81 [1.38; 2.37]
Omitting Taleghani F, 2010	1.77 [1.36; 2.31]
Omitting Thapa S, 2016	1.79 [1.37; 2.36]
Omitting Wang Y, 2007	1.70 [1.31; 2.21]
**Total (95% CI)**	**1.83 [1.40; 2.38]**

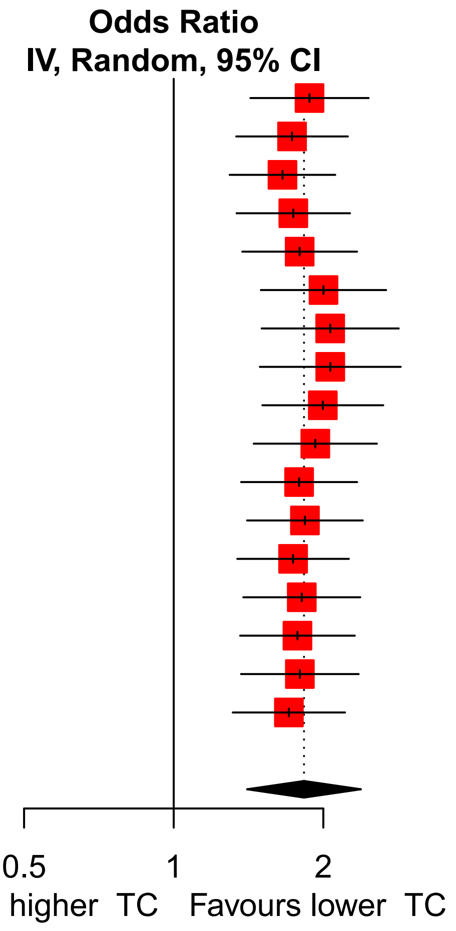

(b) TG

Study	Odds Ratio IV, Random, 95% CI
Omitting Cury EZ, 2018	1.61 [1.34; 1.94]
Omitting Cutler CW, 1999	1.51 [1.27; 1.79]
Omitting Doraiswamy S, 2017	1.49 [1.26; 1.77]
Omitting Golpasand HL, 2014	1.51 [1.27; 1.81]
Omitting Hamissi J, 2011	1.59 [1.32; 1.91]
Omitting Han SJ, 2019	1.71 [1.38; 2.10]
Omitting Kim SR, 2020	1.66 [1.34; 2.05]
Omitting Kushiyama M, 2009	1.65 [1.35; 2.00]
Omitting Lee JB, 2013	1.73 [1.39; 2.15]
Omitting Lee S,2018 (Men)	1.67 [1.37; 2.03]
Omitting Lee S,2018 (Women)	1.63 [1.34; 1.98]
Omitting Losche W, 2000	1.53 [1.28; 1.82]
Omitting Machado AC, 2005	1.56 [1.30; 1.88]
Omitting Moeintaghavi A, 2005	1.52 [1.27; 1.81]
Omitting Monteiro AM, 2009	1.54 [1.29; 1.85]
Omitting Saxlin T, 2008	1.65 [1.36; 2.02]
Omitting Shimazaki Y, 2007	1.57 [1.30; 1.89]
Omitting Taleghani F, 2010	1.57 [1.31; 1.89]
Omitting Wang Y, 2007	1.56 [1.30; 1.88]
**Total (95% CI)**	**1.58 [1.32; 1.90]**

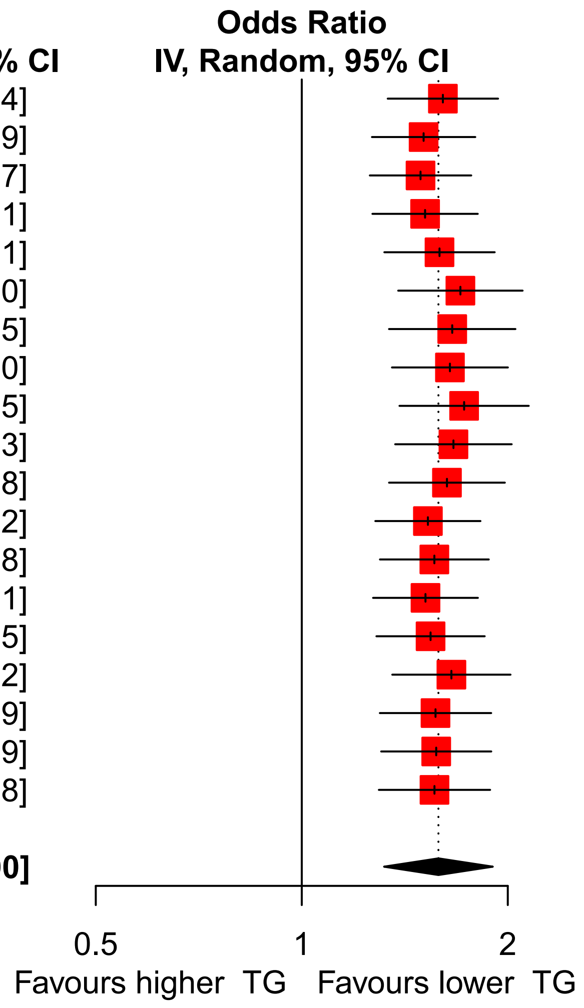

(c) LDL

Study	Odds Ratio IV, Random, 95% CI
Omitting Cury EZ, 2018	1.92 [1.19; 3.09]
Omitting Doraiswamy S, 2017	1.68 [1.15; 2.47]
Omitting Golpasand HL, 2014	2.17 [1.32; 3.57]
Omitting Han SJ, 2019	2.60 [1.36; 4.97]
Omitting Lee S,2018 (Men)	2.47 [1.45; 4.19]
Omitting Lee S,2018 (Women)	2.10 [1.26; 3.52]
Omitting Losche W, 2000	1.92 [1.18; 3.10]
Omitting Machado AC, 2005	2.03 [1.25; 3.27]
Omitting Moeintaghavi A, 2005	2.17 [1.33; 3.54]
Omitting Monteiro AM, 2009	2.03 [1.24; 3.32]
Omitting Saxlin T, 2008	2.62 [1.44; 4.77]
**Total (95% CI)**	**2.11 [1.32; 3.40]**

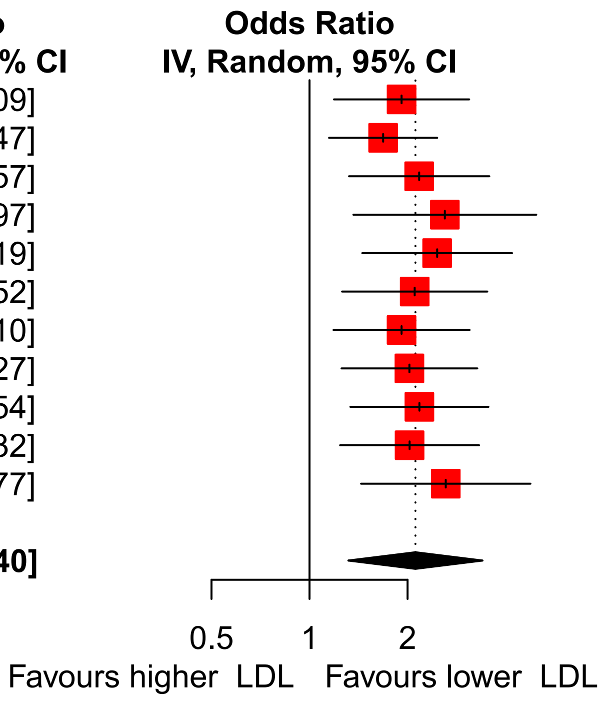

(d) HDL

Study	Odds Ratio IV, Random, 95% CI
Omitting Cury EZ, 2018	1.24 [1.15; 1.35]
Omitting Doraiswamy S, 2017	1.24 [1.15; 1.34]
Omitting Golpasand HL, 2014	1.25 [1.15; 1.35]
Omitting Han SJ, 2019	1.26 [1.14; 1.38]
Omitting Kushiyama M, 2009	1.24 [1.14; 1.34]
Omitting Lee JB, 2013	1.31 [1.19; 1.45]
Omitting Lee S, 2018 (Men)	1.24 [1.15; 1.35]
Omitting Lee S, 2018 (Women)	1.24 [1.14; 1.34]
Omitting Losche W, 2000	1.25 [1.15; 1.35]
Omitting Machado AC, 2005	1.25 [1.16; 1.35]
Omitting Moeintaghavi A, 2005	1.25 [1.15; 1.35]
Omitting Monteiro AM, 2009	1.24 [1.15; 1.35]
Omitting Saxlin T, 2008	1.24 [1.15; 1.35]
Omitting Shimazaki Y, 2007	1.23 [1.14; 1.33]
Omitting Taleghani F, 2010	1.25 [1.15; 1.35]
**Total (95% CI)**	**1.25 [1.15; 1.35]**

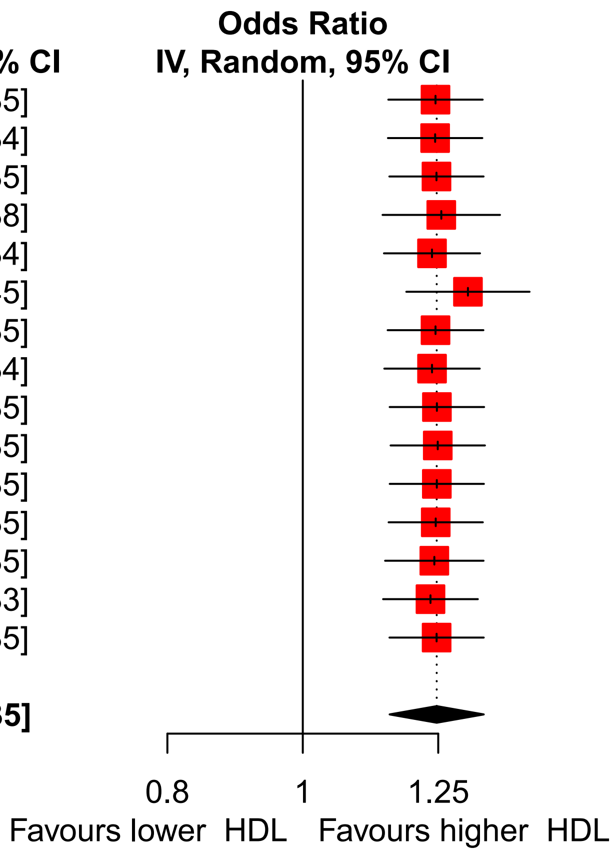

Supplementary Figure 2. Sensitivity analysis of OR values for different lipids levels with the risk of periodontitis. (a) TC; (b) TG; (c) LDL; (d) HDL. Sensitivity analyses were conducted using the leave-one-out method, which removes one study each time and repeats the analysis. The results were robust regardless if any one study was omitted. TC: Total cholesterol, TG: triglycerides, LDL: low-density lipoprotein, HDL: high-density lipoprotein
