# Supplementary material for: Exploring the bi-directional relationship between periodontitis and dyslipidemia: a comprehensive systematic review and meta-analysis
Source: BMC Oral Health. 2024 Apr 29;24:508. doi: 10.1186/s12903-023-03668-7 (PMC11059608; doi:10.1186/s12903-023-03668-7)
Supplement: Supplementary file 10 — Additional file 10. [file 12903_2023_3668_MOESM10_ESM.pdf]

(a) Hyper TG

Study	Odds Ratio IV, Random, 95% CI
Omitting D'Aiuto F, 2008(moderate)	1.21 [1.07; 1.37]
Omitting D'Aiuto F, 2008(severe)	1.19 [1.04; 1.37]
Omitting Fukui N, 2012	1.11 [0.87; 1.42]
Omitting Yu Z,2012	1.16 [1.03; 1.30]
**Total (95% CI)**	**1.17 [1.04; 1.33]**

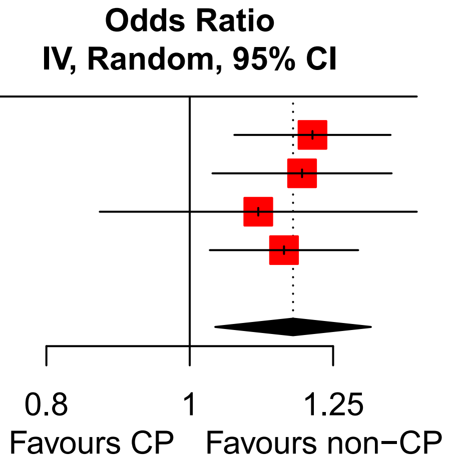

(b) Low HDL

Study	Odds Ratio IV, Random, 95% CI
Omitting D'Aiuto F, 2008(moderate)	1.39 [1.19; 1.63]
Omitting D'Aiuto F, 2008(severe)	1.41 [1.23; 1.61]
Omitting Fukui N, 2012	1.41 [1.17; 1.69]
Omitting Yu Z,2012	1.38 [1.21; 1.58]
Omitting Zhu, HH, 2022	1.42 [1.24; 1.62]
**Total (95% CI)**	**1.40 [1.23; 1.60]**

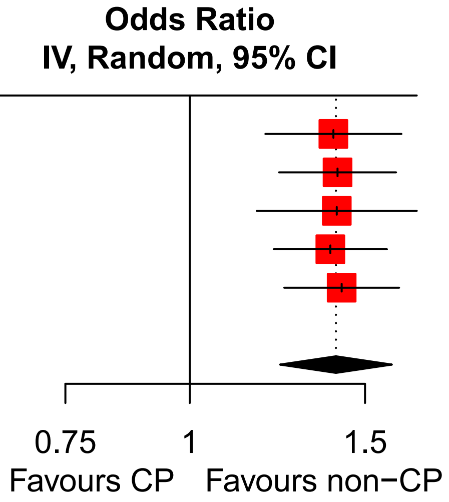

Supplementary Figure 3. Sensitivity analysis of OR values for periodontitis with the risk of dyslipidemia. (a) hyper TG; (b) low HDL. Sensitivity analyses were conducted using the leave-one-out method, which removes one study each time and repeats the analysis. The results were robust regardless if any one study was omitted. TG: triglycerides, HDL: high-density lipoprotein, CP: Chronic Periodontitis
