# Supplementary material for: Exploring the bi-directional relationship between periodontitis and dyslipidemia: a comprehensive systematic review and meta-analysis
Source: BMC Oral Health. 2024 Apr 29;24:508. doi: 10.1186/s12903-023-03668-7 (PMC11059608; doi:10.1186/s12903-023-03668-7)
Supplement: Supplementary file 11 — Additional file 11. [file 12903_2023_3668_MOESM11_ESM.pdf]

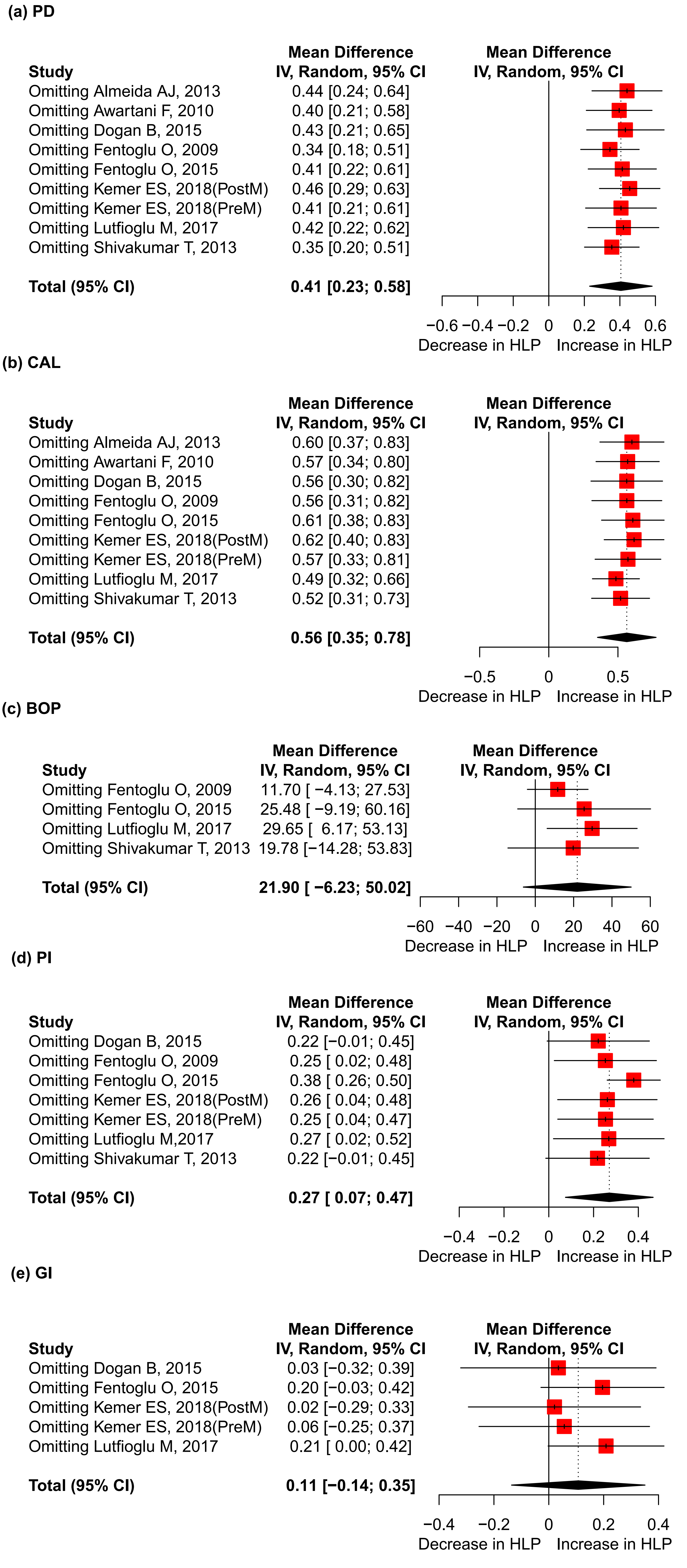

Supplementary Figure 4. Sensitivity analysis of mean difference for comparisons: dyslipidemia versus non-dyslipidemia. (a) PD; (b) CAL (c) BOP; (d) PI; (e) GI. Sensitivity analyses were conducted using the leave-one-out method, which removes one study each time and repeats the analysis. The results were robust regardless if any one study was omitted. PD: probing depth, CAL: clinical attachment level, BOP: bleeding on probing, PI: plaque index, GI: gingival index
