# Supplementary material for: Exploring the bi-directional relationship between periodontitis and dyslipidemia: a comprehensive systematic review and meta-analysis
Source: BMC Oral Health. 2024 Apr 29;24:508. doi: 10.1186/s12903-023-03668-7 (PMC11059608; doi:10.1186/s12903-023-03668-7)
Supplement: Supplementary file 12 — Additional file 12. [file 12903_2023_3668_MOESM12_ESM.pdf]

(a1) TC − baseline

Study	Mean Difference IV, Random, 95% CI
Omitting Fu YW, 2016	9.20 [−0.26; 18.67]
Omitting Oz SG, 2007	4.95 [−5.58; 15.48]
Omitting Tawfig A, 2015	3.65 [−4.84; 12.14]
**Total (95% CI)**	**5.63 [−1.67; 12.93]**

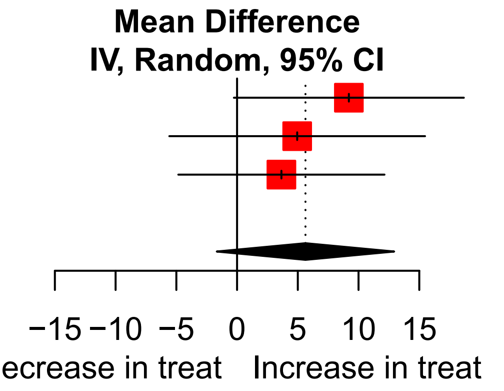

(a2) TC − after 3 months

Study	Mean Difference IV, Random, 95% CI
Omitting Fu YW, 2016	−9.59 [−21.08; 1.90]
Omitting Oz SG, 2007	−6.60 [−15.99; 2.79]
Omitting Tawfig A, 2015	−9.29 [−19.12; 0.55]
**Total (95% CI)**	**−8.32 [−16.59; −0.05]**

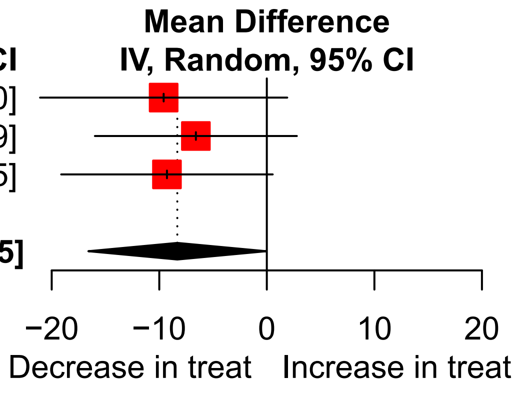

(b1) TG − baseline

Study	Mean Difference IV, Random, 95% CI
Omitting Fu YW, 2016	−15.62 [−49.69; 18.46]
Omitting Oz SG, 2007	−13.00 [−44.98; 18.97]
Omitting Tawfig A, 2015	3.42 [−18.62; 25.46]
**Total (95% CI)**	**−8.61 [−31.62; 14.39]**

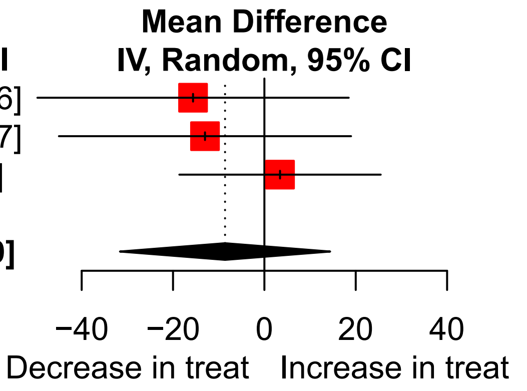

(b2) TG − after 3 months

Study	Mean Difference IV, Random, 95% CI
Omitting Fu YW, 2016	−32.17 [−62.53; −1.81]
Omitting Oz SG, 2007	−37.98 [−56.21; −19.74]
Omitting Tawfig A, 2015	−35.60 [−55.88; −15.32]
**Total (95% CI)**	**−36.13 [−53.63; −18.62]**

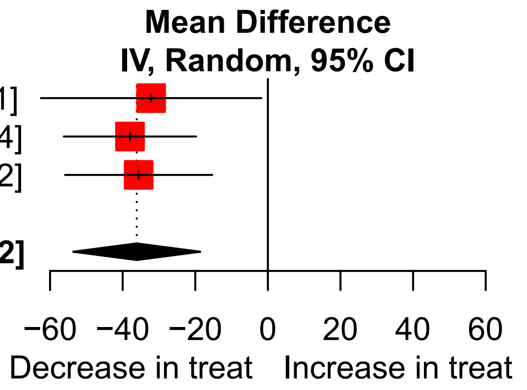

(c1) HDL − baseline

Study	Mean Difference IV, Random, 95% CI
Omitting Fu YW, 2016	−0.46 [−4.69; 3.76]
Omitting Oz SG, 2007	−0.09 [−2.82; 2.63]
Omitting Tawfig A, 2015	−0.10 [−2.81; 2.61]
**Total (95% CI)**	**−0.16 [−2.63; 2.32]**

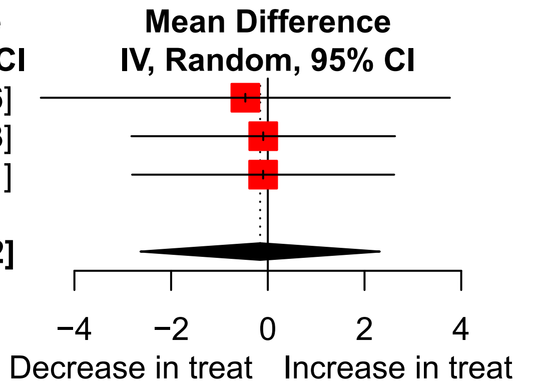

(c2) HDL − after 3 months

Study	Mean Difference IV, Random, 95% CI
Omitting Fu YW, 2016	3.16 [−1.42; 7.74]
Omitting Oz SG, 2007	4.29 [1.84; 6.73]
Omitting Tawfig A, 2015	3.92 [1.52; 6.32]
**Total (95% CI)**	**3.98 [1.71; 6.25]**

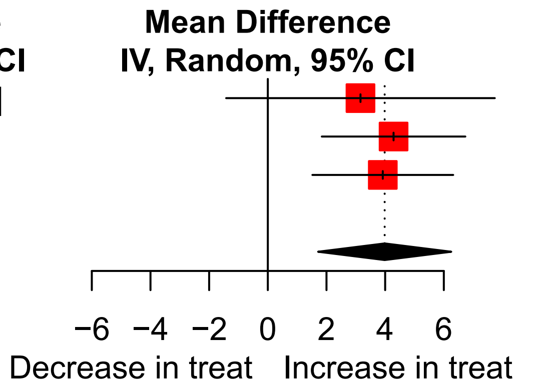

(d1) LDL − baseline

Study	Mean Difference IV, Random, 95% CI
Omitting Fu YW, 2016	9.57 [1.57; 17.57]
Omitting Oz SG, 2007	6.47 [−2.40; 15.33]
Omitting Tawfig A, 2015	6.42 [−0.75; 13.58]
**Total (95% CI)**	**7.46 [0.99; 13.93]**

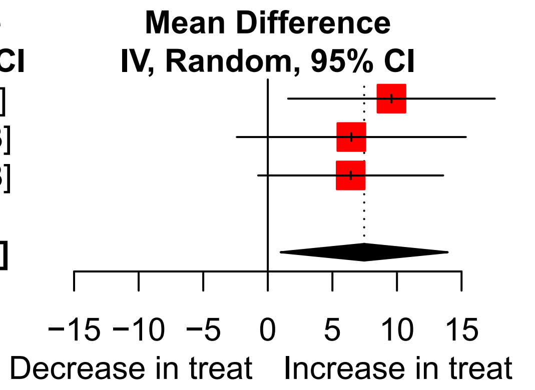

(d2) LDL − after 3 months

Study	Mean Difference IV, Random, 95% CI
Omitting Fu YW, 2016	−25.40 [−41.01; −9.79]
Omitting Oz SG, 2007	−4.84 [−13.73; 4.05]
Omitting Tawfig A, 2015	−14.79 [−36.75; 7.16]
**Total (95% CI)**	**−13.99 [−31.83; 3.86]**

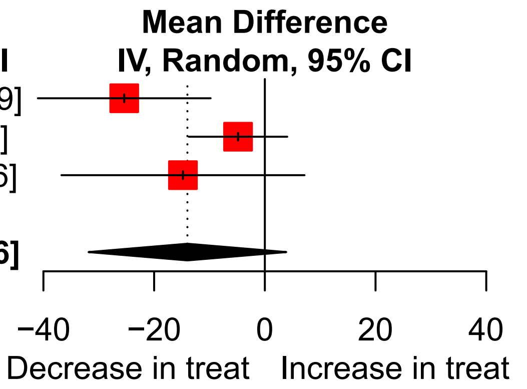

Supplementary Figure 5. Sensitivity analysis of mean difference for comparisons: periodontal treatment versus non-treatment among periodontitis patients. (a1-a2) TC; (b1-b2) TG; (c1-c2) HDL; (d1-d2) LDL. Sensitivity analyses were conducted using the leave-one-out method, which removes one study each time and repeats the analysis. The results were robust regardless if any one study was omitted for TC and TG comparisons. Abbreviations: TC: Total cholesterol, TG: triglycerides, HDL: high-density lipoprotein, LDL: low-density lipoprotein
