# Supplementary material for: Exploring the bi-directional relationship between periodontitis and dyslipidemia: a comprehensive systematic review and meta-analysis
Source: BMC Oral Health. 2024 Apr 29;24:508. doi: 10.1186/s12903-023-03668-7 (PMC11059608; doi:10.1186/s12903-023-03668-7)
Supplement: Supplementary file 13 — Additional file 13. [file 12903_2023_3668_MOESM13_ESM.pdf]

(a) PD

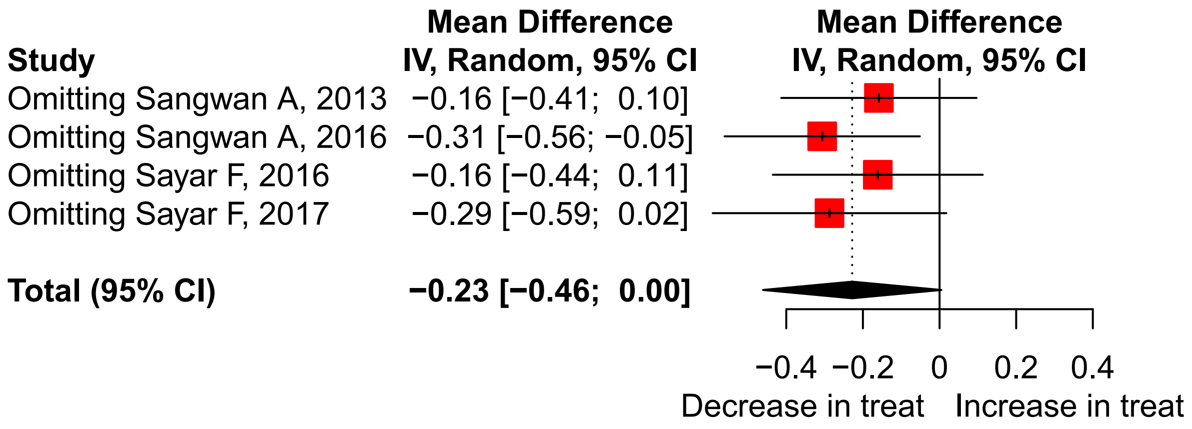

(b) CAL

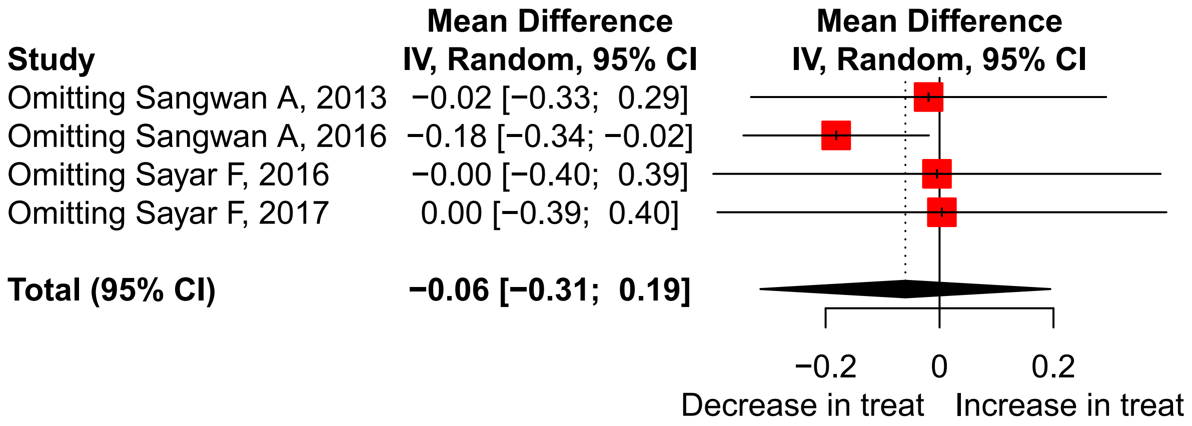

(c) PI

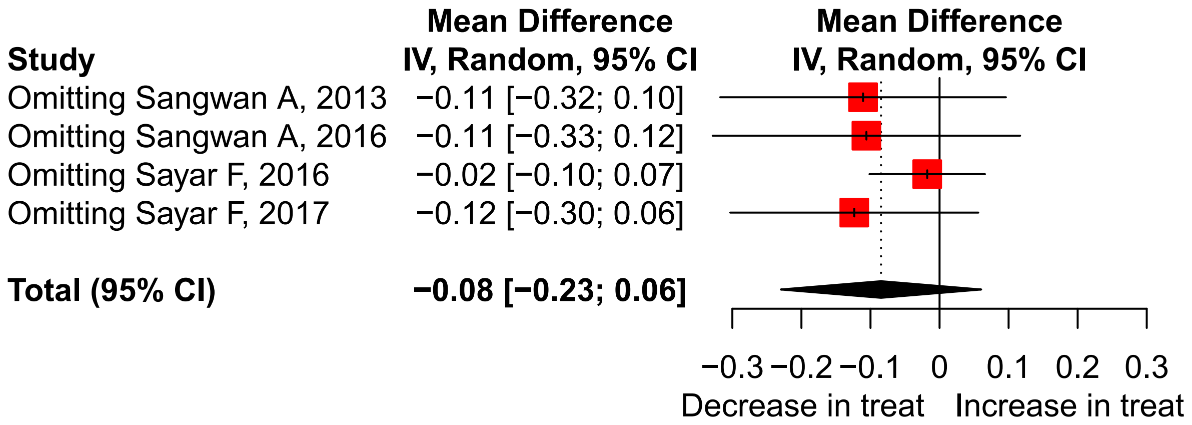

Supplementary Figure 6. Sensitivity analysis of mean difference for comparisons: lipid-lowering treatment versus non-treatment among hyperlipidemia patients. (a)PD; (b)CAL; (c)PI. Sensitivity analyses were conducted using the leave-one-out method, which removes one study each time and repeats the analysis. The results were robust regardless if any one study was omitted for PI comparison. PD: probing depth, CAL: clinical attachment level, PI: plaque index
